# A 3D stem diameter measurement method for field maize at jointing stage: combining RLRSA-PointNet++ and structural feature fitting

**DOI:** 10.3389/fpls.2025.1724096

**Published:** 2026-01-12

**Authors:** Jing Zhou, Yijia Tang, Mingren Cui, Wenlong Zou, Yudi Gao, Yushan Wu, Min Wu, Bowen Jiang, Zhenghong Zhong, Yujie Zou, Lixin Hou, Haijuan Tian

**Affiliations:** 1 College of Information Technology, Jilin Agricultural University, Changchun, China; 2 A Jilin Province Key Laboratory of Grain and Oil Processing, Jilin Business and Technology College, Changchun, China

**Keywords:** maize stem diameter, PointNet++, semantic segmentation, structural feature fitting, three-dimensional point cloud

## Abstract

**Introduction:**

In precision agriculture, accurate measurement of maize stem diameter during the jointing stage is crucial for lodging resistance assessment and yield prediction. However, existing methods have certain limitations: manual measurement is time-consuming and highly subjective, while two-dimensional image recognition can only capture local features and fails to reconstruct the true three-dimensional structure of the stem. Therefore, there is a critical need for an accurate and automated three-dimensional stem diameter measurement approach.

**Methods:**

This study proposes a three-dimensional stem diameter measurement method that integrates an improved PointNet++ segmentation network with structural feature fitting, focusing on the position of the second above-ground internode of maize plants. Specifically, multi-view image reconstruction is employed to generate three-dimensional point clouds of maize stems, and Relative Position Encoding, the Local Group Rearrangement Module, and the Local Region Self-Attention mechanism are incorporated into the PointNet++ network to achieve precise segmentation of stems from the ground. On this basis, a structural feature fitting strategy is applied, where principal axis analysis and ellipse fitting are utilized to extract cross-sectional features, thereby obtaining the major axis and minor axis parameters for stem diameter estimation.

**Results:**

Experimental results demonstrate that the proposed method maintains high accuracy under complex field conditions, achieving a mean absolute error (MAE) of 1.27 mm (R² = 0.87) for major-axis stem diameter and 1.38 mm (R² = 0.82) for minor-axis stem diameter.

**Discussion:**

The proposed method effectively overcomes the limitations of traditional manual and two-dimensional measurement techniques. It provides a robust and accurate solution for maize stem diameter measurement during the jointing stage. This approach offers technical support for intelligent maize growth monitoring, lodging resistance analysis, and three-dimensional phenotypic trait extraction.

## 1 Introduction

Maize is one of the world’s major cereal crops, and its yield and quality are of great importance for ensuring national food security and promoting sustainable agricultural development (Tanumihardjo et al., 2020). The jointing stage is a key stage in the transition from vegetative growth to reproductive growth in the growth process of maize (Fournier and Andrieu, 2000). At this stage, the plants grow rapidly and the internodes elongate quickly. The stem diameter at this stage not only reflects the growth and health of the maize, but is also closely related to its resistance to lodging (Guo et al., 2021). Previous studies have pointed out that thicker stems usually have greater mechanical strength and can effectively support the weight of the ears, thereby reducing the risk of lodging in the middle and late stages (Robertson et al., 2017). Therefore, accurately measuring maize stem diameter during the jointing stage is of great significance for real-time monitoring of crop growth and health, assessing lodging resistance risk, and providing reliable data support for subsequent field management, variety breeding, and precision agriculture (Guan et al., 2020).

In practical agricultural production, traditional measurement of maize stem diameter is mostly carried out manually, such as using a vernier caliper for contact-based measurement. Although such methods are straightforward and easy to implement, they suffer from low efficiency, strong operator subjectivity, and destructive effects on the plants, making them unsuitable for large-scale field measurements and high-throughput data acquisition (Baweja et al., 2017). Additionally, manual measurements can only obtain limited local information from a few points, failing to comprehensively reflect the three-dimensional characteristics of plant structure. This makes it difficult to meet the demands of precision agriculture for efficient, non-contact, and automated monitoring.

To achieve non-destructive and efficient measurement of stem diameter, some studies have introduced two-dimensional vision methods based on image processing. Zhou et al. (2023b) proposed a maize stem extraction algorithm based on HSV color space segmentation and adaptive Otsu thresholding, which achieves non-contact stem diameter estimation through pixel width and reference plate calibration. Additionally, Zhou et al. (2024) developed an internal gradient image segmentation algorithm and a spatial projection method that integrates RGB-D data, further improving measurement accuracy. However, the extraction results of such methods are typically local two-dimensional widths, lacking three-dimensional structural integrity, which limits the accuracy and versatility of parameter modeling.

With the development of three-dimensional reconstruction and sensor technologies, plant structure modeling methods based on point clouds have become an important approach for automated extraction of crop phenotypic parameters. Currently, commonly used techniques for acquiring three-dimensional point clouds include LiDAR (Light Detection and Ranging), Time-of-Flight (ToF) cameras, Structured Light, and Multi-View Stereo (MVS). LiDAR is an active sensor that obtains the three-dimensional coordinates of an object’s surface by emitting laser pulses and measuring their return time (Sakib, 2022). It has high accuracy and high anti-interference capabilities and has been widely used in the macro-structural reconstruction of field crops (Guo et al., 2020a). For example, Wang et al. (2018) used a ground-based laser scanning system to reconstruct a three-dimensional model of an entire maize plant, achieving accurate extraction of structural traits such as plant height and leaf angle. ToF cameras calculate depth based on the propagation time of light pulses, offering advantages such as strong real-time performance and fast data acquisition speed, but their accuracy is limited in outdoor complex lighting conditions and large-scale scenes (He and Chen, 2019). Structured light technology estimates the shape of target surfaces by analyzing the deformation of projected light patterns, making it suitable for close-range high-precision measurements and commonly used in indoor plant phenotyping research (Li et al., 2025). Compared with the above methods, MVS technology does not require an active light source, relying only on RGB cameras to obtain multi-angle images and generating high-quality 3D point clouds through feature matching and dense reconstruction (Wang et al., 2024). Li et al. (2022b) used the MVS method to perform multi-angle image acquisition and 3D reconstruction of maize seedlings, successfully extracting the spatial morphology of the stem and leaf structure. Especially in close-range organ-scale measurement scenarios, MVS can utilize the rich texture information in images to achieve detailed reconstruction (Luo et al., 2024). Its texture expressiveness and geometric accuracy are superior to LiDAR, making it particularly suitable for precise modeling and structural fitting of local structures such as internodes and stems. In actual field applications, although various sensors have made progress to varying degrees, the point clouds collected often suffer from issues such as uneven density, background interference, and structural occlusion, increasing the difficulty of subsequent geometric modeling and phenotypic parameter extraction. Therefore, there is an urgent need to introduce point cloud processing strategies with stronger structural modeling capabilities and context-aware mechanisms to enhance the accuracy of phenotypic parameter extraction and system robustness in complex environments.

To address the challenges posed by the complexity of point cloud data, researchers have gradually introduced deep learning methods into the tasks of structural recognition and semantic segmentation of plant 3D point clouds (Guo et al., 2020b). Among these, the PointNet++ network has emerged as the current mainstream architecture for plant point cloud analysis due to its ability to directly process unordered raw point clouds and extract multi-scale features (Qi et al., 2017). Guo et al. (2023) introduced an improved PointNet++ network in organ-level point cloud segmentation of cabbage, achieving a classification accuracy of 95% and an intersection-over-union (IoU) of 86%. Turgut et al. (2022) incorporated an attention mechanism into the original framework, significantly enhancing point-to-point dependency modeling capabilities, resulting in a 4% improvement in mIoU for rose plant point cloud segmentation tasks. Additionally, Du et al. (2023) proposed the Plant Segmentation Transformer (PST) based on the Transformer architecture, achieving 93.96% mIoU and 97.07% overall accuracy (OA) in rapeseed point cloud segmentation experiments. Although these methods have made significant progress in organ-level segmentation, they still suffer from insufficient structural modeling and limited semantic expression in typical field scenarios characterized by complex structures, uneven density, and background interference, which affects the accuracy and robustness of point cloud segmentation. For three-dimensional measurement of maize stem diameter, Miao et al. (2022) proposed a method for measuring the morphological parameters of maize in the field based on point cloud image conversion. They used terrestrial laser scanning (TLS) to obtain maize point clouds at multiple growth stages and automatically extracted plant height, stem diameter, and short axis through steps such as denoising, segmentation, and ellipse fitting. This method showed good adaptability and accuracy for different varieties and growth stages. However, lidar equipment is costly and prone to obstruction in complex field environments, limiting its large-scale application and ability to reconstruct detailed structures. This indicates that current point cloud measurement methods still have shortcomings in terms of robustness and fine modeling.

Although 3D point cloud technology has made significant progress in the field of plant phenotyping, it still faces numerous challenges in actual field environments. Due to high plant density and the similar morphology and color of adjacent plants, point cloud data is prone to aliasing and occlusion, making it difficult to accurately segment individual plant boundaries (Wang et al., 2020). Therefore, effectively integrating the geometric features of 3D point clouds with the spatial distribution patterns of plant organs, and optimizing point cloud data processing and feature extraction strategies, has become a key factor in improving the accuracy and automation of phenotype parameter extraction. Addressing these issues, designing targeted feature extraction methods to enhance structural perception and contextual understanding is an urgent research priority that requires immediate attention.

The main innovations of this paper are as follows:

1. To address the complexity of maize stem structures in field conditions and the limited accuracy of traditional measurement methods, which can only capture local phenotypic traits, we propose a 3D stem diameter measurement method that integrates the RLRSA-PointNet++ network with structural feature fitting. This method enables high-precision modeling and measurement of key internode regions during the maize jointing stage, allowing the acquisition of both major and minor axes of stem diameter.
2. To address the limitations of point cloud structural modeling and restricted local semantic expression, we developed an improved semantic segmentation network that integrates Relative Position Encoding, Local Reordering, and Local Region Self-Attention mechanisms, effectively enhancing the robustness and accuracy of point cloud semantic recognition.
3. To address the difficulty of obtaining complete geometric parameters using conventional image-based methods, we propose a stem diameter calculation method based on principal axis extraction and cross-sectional elliptical fitting with structural feature modeling, enabling automatic computation of both the major and minor stem diameters.

The proposed method provides an automated and high-precision solution for maize stem diameter measurement, which can promote the development of in-field crop phenotyping and support precision agriculture practices.

## 2 Materials and methods

### 2.1 Materials

Maize stem samples were collected from the teaching and research base of Jilin Agricultural University, located in Changchun, Jilin Province, China. Data acquisition was conducted between 15:00 and 18:00 in July 2024 under clear weather conditions. The spacing between maize plants was 0.3 m, and the inter-row spacing was 0.6 m. The study focused on the stem segment from the second node above the ground during the jointing stage, which is representative and conducive to structural feature analysis.

### 2.2 Experimental design

**Figure 1:**
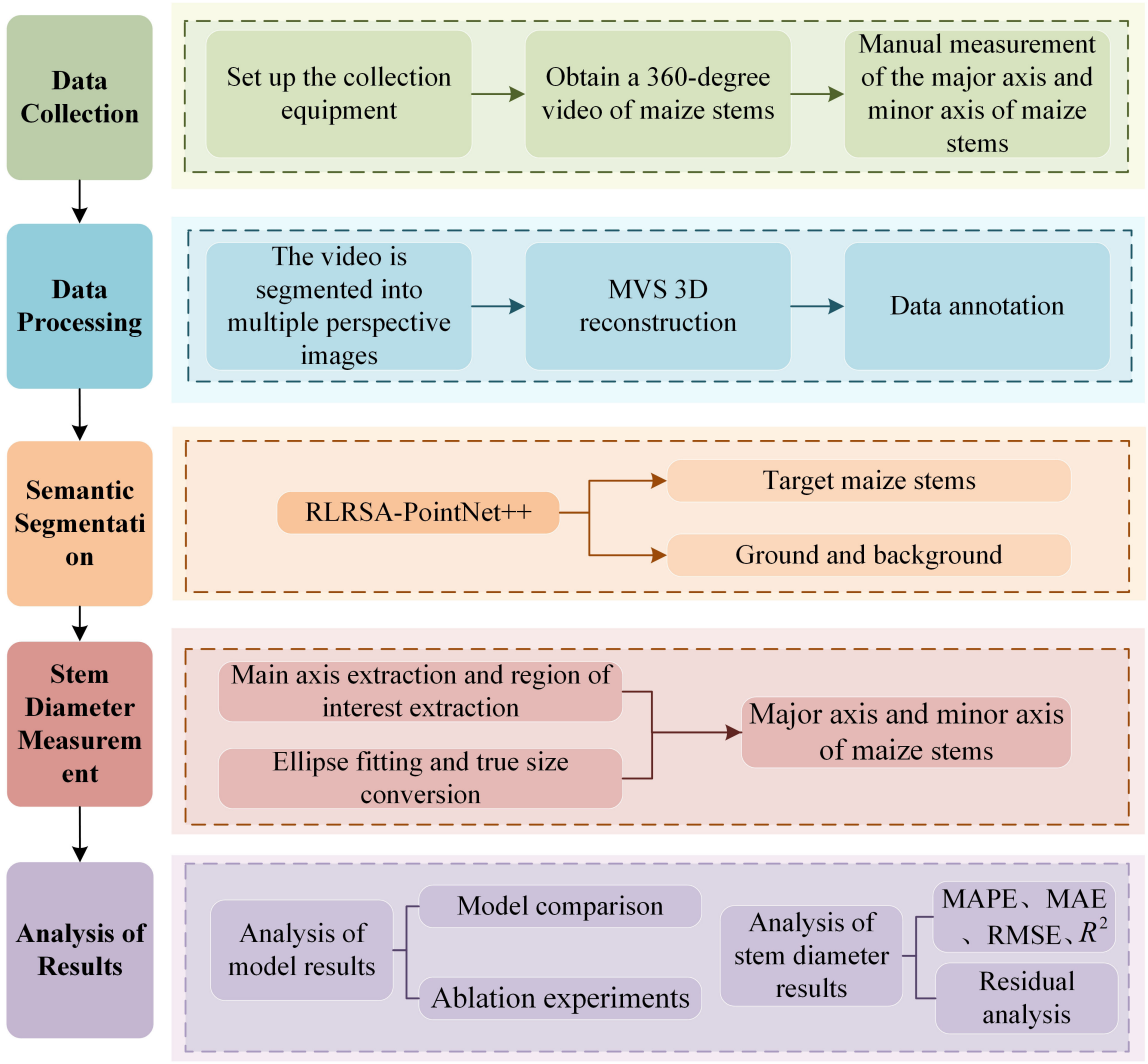
Main workflow of the study.

The research content of our study mainly includes data acquisition, data processing, semantic segmentation of maize stems and ground background, stem diameter measurement, and result analysis. The main work is shown in **Figure 1**.

**Figure 2:**
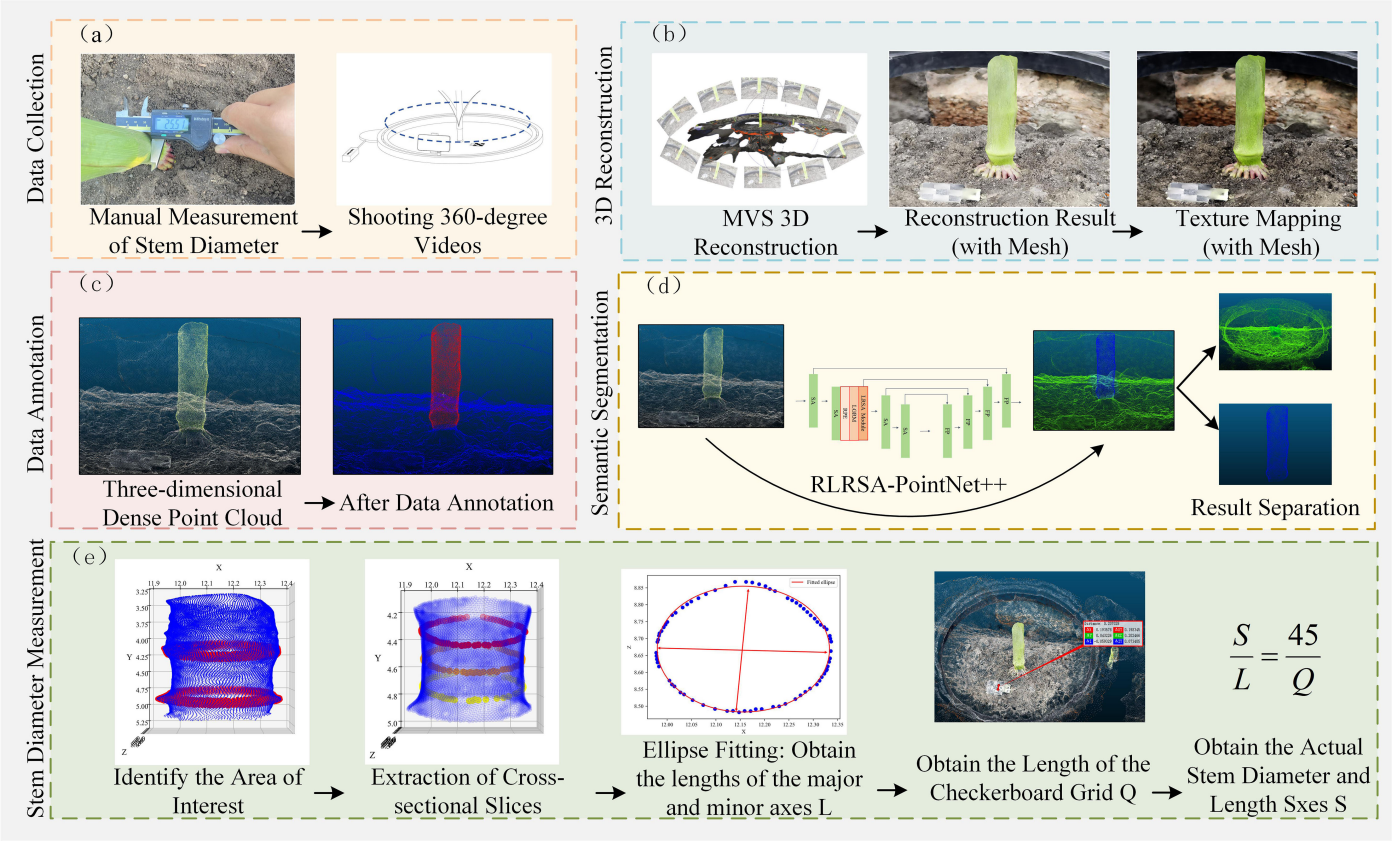
Flowchart of the stem diameter 3D measurement experiment: **(a)** Data Collection, **(b)** 3D reconstruction, **(c)** Data annotation, **(d)** Semantic segmentation, **(e)** Stem diameter measurement.

The three-dimensional measurement of maize stem diameter during the jointing stage in this study consists of five parts: (a) Data Collection, (b) 3D Reconstruction, (c) Data Annotation, (d) Semantic Segmentation, and (e) Stem Diameter Measurement. The overall experimental process is shown in **Figure 2**.

#### 2.2.1 Data collection

The data collection and multi-view stereo reconstruction (MVS) (Furukawa and Hernández, 2015) work described in this paper was completed between July 3 and 10, 2024. To establish accurate ground-truth measurements for validating the proposed 3D stem diameter estimation method, 72 maize samples at the jointing stage were selected under field conditions. The stem diameter at the second internode above the ground was manually measured using a Vernier caliper with a precision of 0.01 mm. To ensure reliability and minimize human-induced variability, measurements were independently conducted by two agronomy-trained experimenters. For each maize sample, researchers collected data at positions perpendicular to the main axis of the stem, measuring the diameter at three uniformly distributed locations. At each measurement point, manual calipers were used to take measurements along two mutually perpendicular directions. One direction measured a longer length, while the perpendicular direction measured a shorter length. Specifically, the direction of maximum diameter corresponded to the major axis of the elliptical stem cross-section, and its perpendicular direction corresponded to the minor axis. Each researcher independently measured each data point. Thus, two researchers measured the major and minor axes at three positions on the same maize stem. Each stem ultimately yields six sets of data. The final true diameter of the sample is determined by averaging the valid measurements from both operators. This manual measurement protocol ensures data reliability and accuracy, providing precise baseline data for evaluating automated diameter extraction methods based on 3D point clouds.

The data collection system primarily consists of a smartphone, a remote-controlled motorized turntable, a three-way adjustable stand, and a portable power bank with a capacity of 2600 mAh. To ensure stable, continuous, and reliable image data acquisition in the field environment, the electric turntable was placed horizontally and secured to the ground during the experiment, while the maize stem samples were fixed vertically at the center of the turntable to ensure consistency and integrity of the viewing angle during rotation.

**Figure 3:**
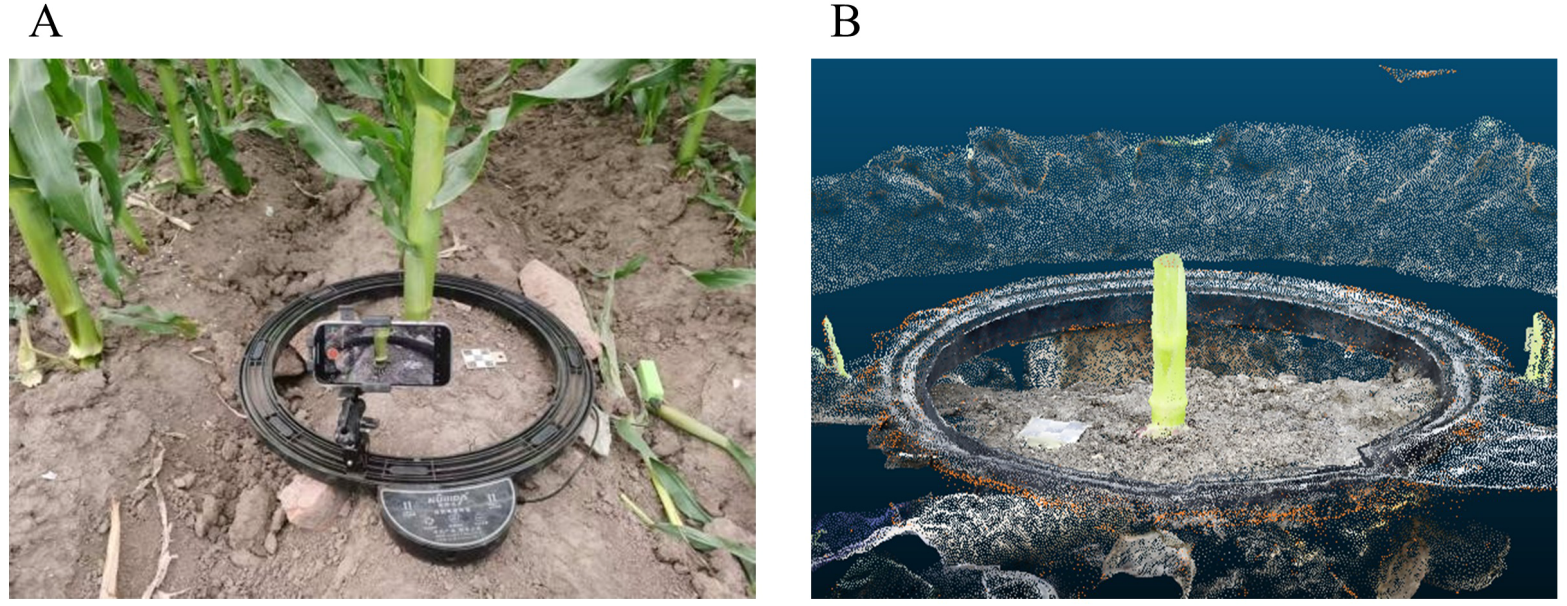
Data collection. **(A)** Data collection device Figure. **(B)** 3D dense point cloud.

Our investigation used a smartphone capable of shooting 4K resolution video as the data collection device. By controlling the rotating platform to rotate at a low and constant speed, the smartphone was able to capture 360° multi-angle images around the maize stems. At the same time, a checkerboard calibration plate was used as a scale reference. During data collection, the checkerboard calibration plate was placed near the maize plants, as shown in **Figure 3A** During data collection, the video recording frame rate was set to 60 frames per second to ensure clear and continuous image sequences, thereby improving the accuracy and integrity of the subsequent 3D reconstruction. Each collection lasted about 90 seconds, comprehensively covering the external structural features of the maize stems and the checkerboard calibration board. Subsequently, key frames were extracted from the video at a time interval of one frame per second to generate an image sequence, which was then input into the multi-view stereo (MVS) algorithm framework to construct a high-density 3D point cloud, providing a data basis for subsequent semantic segmentation and morphological feature extraction.

This study uses an OpenMVS-based multi-view stereo vision method to achieve high-precision 3D reconstruction of maize stems. First, the Structure from Motion (SfM) (Jiang et al., 2020)algorithm is used to extract and match feature points from multi-view images, combine geometric constraints to estimate the external parameters of the camera, and generate a preliminary sparse point cloud through triangulation. To improve the reconstruction accuracy, bundle adjustment is introduced in the SfM stage to jointly optimize the camera parameters and 3D point positions (Wei et al., 2020).Considering that the point cloud obtained in the SfM stage is relatively sparse and difficult to meet the needs of subsequent detailed analysis, the MVS algorithm is further introduced to generate a dense point cloud. This process estimates the depth map of multi-view images based on the camera pose restored by SfM, optimizes the depth accuracy through photometric consistency constraints, and finally fuses to generate a high-resolution dense point cloud. To improve point cloud quality, post-processing operations such as denoising and hole filling are applied, and the complete geometric structure is restored using a triangular mesh generation method (Gonizzi Barsanti et al., 2024).Key parameters for 3D reconstruction are shown in **Table 1**.

**Table 1:** Key parameters for 3D reconstruction.

Stage	Parameter	Actual value
Image acquisition	Number of images per object	90 images
	Image resolution	3840×2160 pixels
Feature extraction	max_image_size	3200
	max_num_features	8192
Feature matching	Matching mode	Sequential
	max_ratio	0.8
	cross_check	Enabled
SfM/Triangulation	min_tri_angle	2–4°
OpenMVS Densify	resolution-level	0
	min_views	3
	patch-match-iterations	5
	consistency-threshold	2
MeshLab (filtering)	Outlier removal neighbors	50
	Outlier removal std	1
MeshLab (resampling)	Poisson disk spacing	0.25–0.5 mm
Export	Coordinates precision	8

The focus of this study is on the semantic analysis of dense point clouds. To reduce the interference of noise on the analysis results during the reconstruction process, MeshLab is used to perform texture mapping and geometric optimization on the generated mesh model, and finally resampling is performed to obtain a high-quality dense point cloud with minimal noise interference. This point cloud provides a stable and reliable data foundation for subsequent semantic segmentation and morphological feature extraction. The final 3D reconstruction of the dense point cloud is shown in **Figure 3B**.

The reconstructed maize stem point clouds exhibit an average density of approximately 389,685 points per sample, indicating a high-resolution 3D representation of structural details. To further characterize the spatial point distribution, voxel-based density analysis was performed with a voxel. The overall average voxel density was calculated as 610.3192 points/voxel, demonstrating sufficient local feature richness for accurate geometry fitting and semantic segmentation.

#### 2.2.2 Data annotation

After texture mapping, the 3D dense point cloud was imported into CloudCompare software (Antova and Peev, 2024) (https://cloudcompare.org/) for manual annotation. During the annotation process, the cropping function provided by CloudCompare was used to select the maize stem area by connecting multiple points, accurately separating the target area from the background information. Subsequently, the “Add constant SF” function was used to assign a label value of 1 to the maize stem area and a label value of 0 to the remaining background area, thereby completing the binary classification annotation of the point cloud data (Liu et al., 2025b).To ensure annotation consistency, two annotators trained in agricultural science independently annotated the point cloud data using identical standards. Concurrently, we implemented a cross-verification mechanism, any discrepancies require joint review and correction by both annotators.

According to the semantic annotations, the dense point cloud is divided into two categories: background (label 0) and maize stem (label 1). Statistical analysis shows that the background region contains an average of approximately 371,306 points, with a mean voxel density of about 587 points per voxel. In contrast, the maize stem region comprises fewer total points (approximately 18,534 on average), but its voxel density reaches approximately 5,006 points per voxel.This substantial difference in spatial density indicates that the maize stem region exhibits a more compact geometric distribution and retains a higher level of structural detail. Such localized high-density characteristics imply richer feature information, which is beneficial for improving the accuracy and robustness of downstream semantic segmentation and morphological measurements.

#### 2.2.3 Semantic segmentation method based on RLRSA-PointNet++

Three-dimensional point cloud data collected from maize stems in complex field environments usually has irregular structures, uneven point density distribution, and severe background interference. These factors significantly increase the difficulty of semantic segmentation, causing the following two problems when using traditional PointNet++-based segmentation methods on this type of data:

1. Limited spatial structure modeling capability. PointNet++ relies on local sampling and multi-scale feature aggregation mechanisms for spatial feature extraction, with its core multi-layer perceptron (MLP) primarily learning features based on the coordinate differences between points (Li et al., 2022a). However, this approach does not incorporate the geometric relationships and relative positional information between points and the central point, resulting in insufficient structural awareness when handling regions with complex structures or significant density gradients, thereby affecting segmentation accuracy.
2. Insufficient local information representation capability. Intermediate-level semantic features often contain rich local geometric shapes and boundary information, which are crucial for object recognition in complex backgrounds. However, the original PointNet++ network lacks the ability to model long-range dependencies between points in intermediate-scale regions, limiting its segmentation performance in scenarios with blurred edges and strong background interference.

To improve the three-dimensional semantic segmentation performance of maize stems and background areas, this paper introduces an improved network termed RLRSA-PointNet++, which is constructed by integrating Relative Position Encoding (RPE), Local Group Rearrangement Module (LGRM), and Local-Region Self-Attention (LRSA) (Liu et al., 2025a) into the original PointNet++ framework. These modules jointly optimize the structural representation of the network, thereby enhancing its capability to model local geometric structures and contextual relationships.

**Figure 4:**
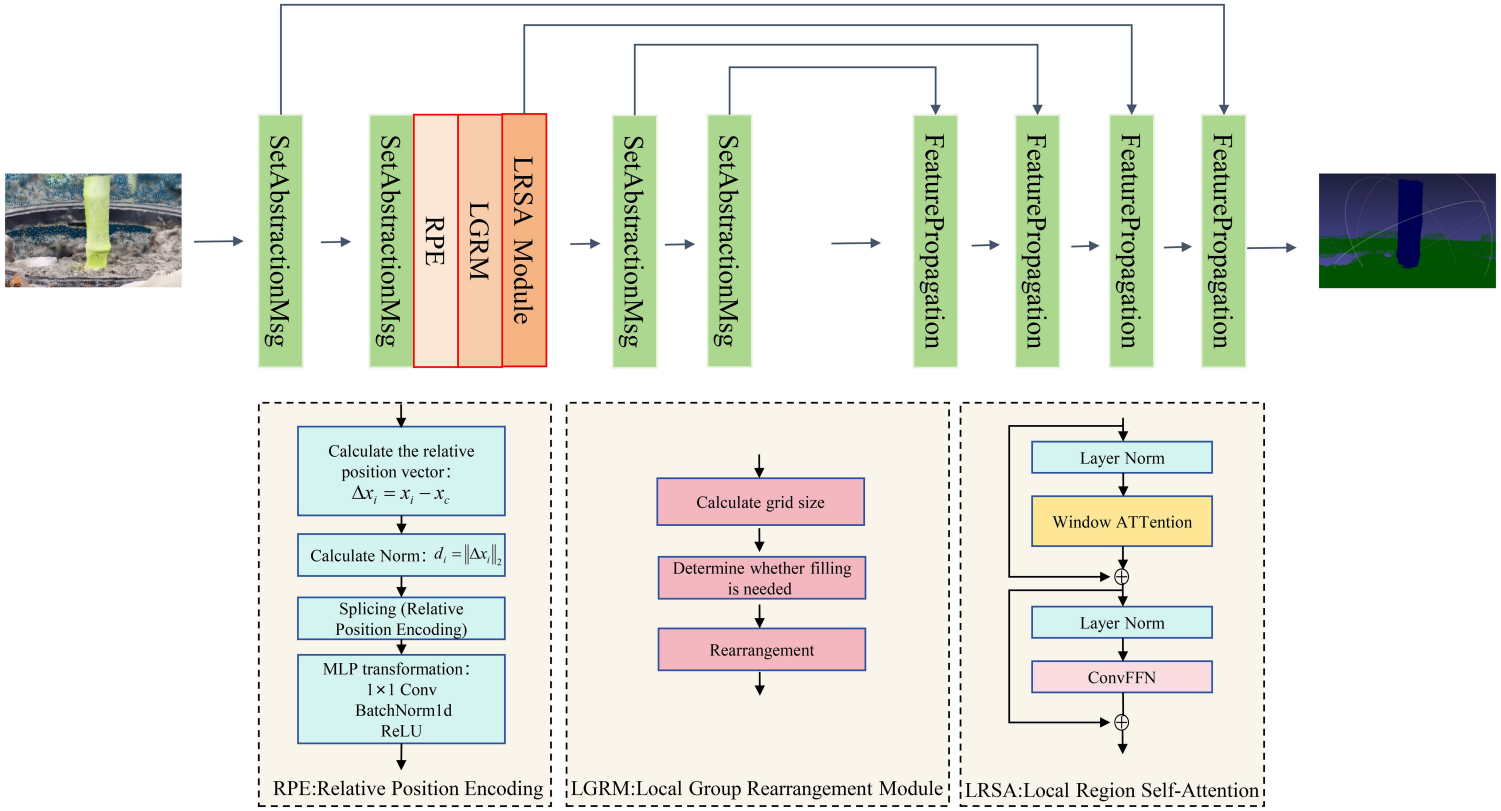
RLRSA PointNet++ network structure figure.

The overall structure of the improved PointNet++ network is shown in **Figure 4**. The input of this method is raw point cloud data, where each batch contains B samples, and each sample consists of N unordered points. Each point has three-dimensional spatial coordinates (x,y,z), represented as a coordinate tensor of shape (B,3,N), and additional descriptive features such as color and normal vectors with a shape of (B,6,N). After concatenation, the original input feature tensor becomes (B,9,N), indicating that each point is described by 9-dimensional attributes. During the feature extraction stage, the network first employs multiple Set Abstraction (SA) modules to progressively downsample the point cloud and aggregate local features. For example, SA1 downsamples the original N points to 1024 keypoints and extracts 64-dimensional features, resulting in coordinate and feature tensors of (B,3,1024) and (B,64,1024), respectively. SA2 further captures higher-level semantic features, reducing the point number to 256 and outputting (B,3,256) and (B,128,256), where 64 and 128 denote the feature channel sizes.

To enhance spatial structure modeling, three local structure enhancement modules are introduced. First, RPE embeds the relative geometric relationships between each local point and its center, maintaining the feature dimension (B,128,256). Then, the LGRM reorganizes unordered point features into a structured 2D grid, transforming the feature into (B,128,16,16), where 16×16 = 256 represents the spatial projection of local regions. Next, LRSA applies a self-attention mechanism on the structured grid to capture long-range dependencies within local regions, keeping the tensor shape unchanged. Afterward, the grid features are flattened back to point-wise form, recovering to (B,128,256). The SA3 and SA4 modules continue hierarchical feature abstraction, reducing the point number from 256 to 64 and then 16, while increasing feature channels to 256 and 512, producing (B,3,64), (B,256,64), (B,3,16), and (B,512,16), thus achieving multi-level semantic compression and global feature aggregation.

Subsequently, four Feature Propagation (FP) modules perform progressive upsampling and feature fusion, sequentially propagating global semantic features back to the intermediate (64), lower (256), fine-grained (1024), and final full-resolution N points, ultimately generating 128-dimensional features for each original point, with a tensor shape of (B,128,N). Finally, the Head classification module applies point-wise convolution, dropout, and activation to produce point-wise semantic predictions, mapping the features to class probabilities with an output shape of (B,N,*num_classes*), where *num_classes* denotes the total number of semantic categories. Overall, the network achieves a complete pipeline of hierarchical abstraction, relational modeling, and feature reconstruction from raw point clouds to point-wise semantic labels. Here, B represents the batch size, N the number of original points, 3 the spatial coordinate dimension, and 64, 128, 256, 512 indicate feature channel dimensions.As shown *in***Table 2***, Tenso*rFlow interacts with modules within the RLRSA-PointNet++ architecture.For more detailed information, please refer to **Supplementary Material 1**.

**Table 2:** Tensor flow and module interaction in RLRSA-PointNet++ architecture.

Stage	Input coordinates	Input features	Conceptual input format	Module	Output tensor	Description
L0	(B,3,N)	(B,6,N)	(B,3,N)+(B,6,N)	Raw Input	(B,9,N)	Raw geometry and appearance data
SA1	(B,3,N)	(B,9,N)	(B,3,N)+(B,9,N)	Set Abstraction	(B,3,1024),(B,64,1024)	Local geometry & appearance extraction
SA2	(B,3,1024)	(B,64,1024)	(B,3,1024)+(B,64,1024)	Set Abstraction	(B,3,256),(B,128,256)	Enhanced structural and semantic encoding
RPE	(B,3,256)+(B,3,1)	(B,128,256)	(B,128,256)	Relative Position Encoding	(B,128,256)	Encodes spatial relation to local center
LGRM	-	(B,128,256)	-	Local Group Rearrangement Module	(B,128,16,16)	Reorders unordered points into structured grid
LRSA	-	(B,128,16,16)	-	Local-Region Self-Attention	(B,128,16,16)	Captures local region long-range dependencies
Flatten	-	(B,128,16,16)	-	Reshape	(B,128,256)	Restores point-wise structure
SA3	(B,3,256)	(B,128,256)	(B,3,256)+(B,128,256)	Set Abstraction	(B,3,64),(B,256,64)	Higher-level semantic abstraction
SA4	(B,3,64)	(B,256,64)	(B,3,64)+(B,256,64)	Set Abstraction	(B,3,16),(B,512,16)	Global semantic aggregation
FP4	(B,3,64)+(B,3,16)	(B,256,64)+(B,512,16)	-	Feature Propagation	(B,256,64)	Propagate global to mid-level
FP3	(B,3,256)+(B,3,64)	(B,128,256)+(B,256,64)	-	Feature Propagation	(B,256,256)	Fuses mid- and high-level features
FP2	(B,3,1024)+(B,3,256)	(B,64,1024)+(B,256,256)	-	Feature Propagation	(B,128,1024)	Refines point-wise features
FP1	(B,3,N)+(B,3,1024)	None+(B,128,1024)	-	Feature Propagation	(B,128,N)	Full-resolution feature recovery
Head	-	(B,128,N)	-	Conv+Dropout	(B, N, num_classes)	Point-wise semantic prediction

1. Relative Position Encoding (RPE):

This module explicitly encodes the three-dimensional relative positions and Euclidean distances of neighborhood points relative to the cluster centers, guiding the network to learn spatial relationships within local geometric structures.

The RPE module is integrated after the second-level set abstraction layer (SA2) based on architectural inference and feature representation analysis. The rationale for this placement is as follows: At the SA1 layer, the focus is primarily on capturing low-level geometric and color features. At this stage, local spatial relationships are unstable and susceptible to noise interference, making it unsuitable for placement after SA1. In contrast to SA1, the highly abstracted semantic features generated after SA3 and SA4 have undergone geometric aggregation, limiting the effectiveness of spatial encoding. SA2, as an intermediate stage, preserves neighborhood-level geometric information while initiating semantic context encoding. This provides the optimal feature space for enhancing local structures via RPE. Furthermore, SA2 outputs 128 channels, matching the designed RPE feature dimension. This enables direct element-wise fusion without additional mapping, enhancing implementation simplicity and reproducibility.

Let the input be the 3D coordinates of a local point set with coordinates set to $X={\left\{x_{i}\right\}}_{i=1}^{N}\in {\mathbb{R}}^{3\times N}$, representing the set of 3D coordinates for all points within the local region. Here, N denotes the number of neighboring points, and the center coordinates of the region are set to $x_{c}\in {\mathbb{R}}^{3}$. The relative position vector for each point is given by Equation 1, where it represents the 3D position difference vector of the i-th point relative to the cluster center, indicating the spatial offset between the point and the center. The 3D relative position includes components $\Delta x_{i},\Delta y_{i},\Delta z_{i}$.

(1) $$\Delta x_{i}=x_{i}-x_{c}\in {\mathbb{R}}^{3}$$

The Euclidean distance is shown in Equation 2, where $d_{i}$ represents the Euclidean distance between the i-th point and the cluster center.

(2) $$d_{i}={\left||\Delta x_{i}|\right|}_{2}\in \mathbb{R}$$

The position encoding vector is constructed as shown in Equation 3, representing the relative position encoding vector of the i-th point, which includes three-dimensional relative position and Euclidean distance, for a total of four dimensions.

(3) $$r_{i}={\left[\text{Δ}x_{i},\text{Δ}y_{i},\text{Δ}z_{i},d_{i}\right]}^{T}\in {\mathbb{R}}^{4}$$

Form a tensor $R\in {\mathbb{R}}^{4\times N}$, which represents the tensor formed by concatenating all $r_{i}$ encoding vectors along the columns, where each column is the relative position encoding of a point, 4 rows correspond to 4-dimensional encoding, and N columns correspond to N points. After a 1×1 convolution, the MLP maps to the target dimension as shown in Equation 4:

(4) $$E=\text{ReLU}\left(\text{BN}\left(W*R\right)\right)\in {\mathbb{R}}^{C\times N}$$

The * denotes a 1×1 convolution operation, where $W\in {\mathbb{R}}^{C\times 4}$ is the convolution kernel, BN represents batch normalization (BatchNorm)(Gao et al., 2021), and ReLU is the activation function. The final relative position features E are added to the original features for enhancement, as shown in Equation 5, where $f_{i}^{'}$ denotes the fused features used to further represent the spatial information and context of the point. $f_{i}$ denotes the original point feature, i.e., the output from the previous layer of the PointNet++ network, and $e_{i}$ denotes the enhanced feature generated by relative position encoding.

(5) $$f_{i}^{'}=f_{i}+e_{i}$$

2. Local Group Rearrangement Module (LGRM):

The inherent disorderliness and irregularity of local structures in point cloud data make it difficult for attention mechanisms to adapt directly. The Local Group Rearrangement Module (LGRM) can map the disordered point features of each local region into an approximate two-dimensional grid structure, providing structured input for subsequent local region self-attention mechanisms.

Let the input point feature be: $F\in {\mathbb{R}}^{C\times N}$, where N is the number of points in the local region and C is the number of channels. The goal is to rearrange it into a network tensor: $G\in {\mathbb{R}}^{C\times h\times w}$, where $h=w=\sqrt{N}$, h and w are the height and width of the two-dimensional feature map after rearrangement. Next, determine whether padding is needed. If N<h×w, use the forward mirror padding strategy to pad the features, as shown in Equation 6, where $F_{\text{padded}}$ denotes the padded feature tensor; P=h×w−N denotes the number of points required to pad the features into a complete two-dimensional grid structure; and F[:,:P] denotes copying the first P point features from the input feature F for mirror padding.

(6) $$F_{\text{padded}}=\left[F,F\left[:,:P\right]\right],P=h\times w-N$$

Finally, reshape obtains a two-dimensional feature map, corresponding to the formula shown in Equation 7, where G represents the output three-dimensional tensor, representing the rearranged two-dimensional grid feature map; $G_{C,:,:}$ represents the two-dimensional feature map on the cth channel of G, that is, the h×w grid corresponding to channel c; F represents the input point feature tensor, representing the C-dimensional features of N points; $F_{c,:}$ denotes the cth row of F, representing the features of all N points in the cth channel; reshape denotes reshaping the input vector into a two-dimensional matrix; c denotes the index of the current channel; C denotes the number of feature channels, i.e., the feature dimension of each point.

(7) $$G_{C,:,:}=reshape\left(F_{c,:}\right)forc=1,\ldots ,C$$

3. Local Region Self-Attention (LRSA) Mechanism.

To enhance the spatial modeling capabilities of mid-level semantic features and the perception capabilities of boundary regions, this paper designs a Local Region Self-Attention (LRSA) that uses local patch division, multi-head attention mechanisms, and deep convolutional feedforward networks (ConvFFN) (Wang et al., 2025) to collaboratively model long-range dependencies in local structures.

The LRSA module is designed to enhance semantic interaction among points within a local neighborhood, addressing the limitations of the original PointNet++ in modeling geometric and relational structures. Once the unordered point features are rearranged into a structured feature map (B, 128, h, h) by LGRM, LRSA performs region-level relational modeling on this structured representation, which fundamentally differs from the per-point MLP-based processing in the standard SA modules.

In LRSA, each rearranged feature block is treated as a spatially structured local relation graph. While keeping the channel dimension fixed at 128, LRSA dynamically refines each point feature by considering dependencies, spatial continuity, and semantic similarity with its neighboring points.

Throughout the process, LRSA maintains the original tensor dimensionality; both input and output remain (B, 128, h, h). After relation-aware enhancement, the features are flattened back to (B, 128, N), allowing seamless integration with SA3 and subsequent Feature Propagation (FP) layers, without altering the overall framework of PointNet++.For the specific formula derivation, please refer to **Supplementary Material 2**.

In summary, the LRSA module significantly improves the network’s ability to model local structural continuity and contextual relevance by performing relation-aware attention on structured neighborhood features. This enhancement provides richer and more discriminative feature representations for downstream semantic segmentation and structural understanding tasks, while keeping the overall architecture reproducible and consistent.

#### 2.2.4 Stem diameter measurement

First, the 3D point cloud output by the RLRSA-PointNet++ network is separated into background and stems. In 3D point cloud data processing, the region of interest (ROI) is the second node of the maize stem from the ground (Zhou et al., 2023a), and the main axis direction of the maize stem is the key basis for determining the region of interest.

Step 1: Stem principal axis extraction and coordinate normalization.

In order to accurately extract the main axis direction of the stem, this paper uses the principal component analysis (PCA) method for feature extraction (Kurita, 2021). PCA is a classic statistical dimension reduction technique that identifies the primary directions of point cloud data distribution by performing feature decomposition on the covariance matrix of the point cloud data (Duan et al., 2021). In our study, the objective of PCA is to determine the principal axis direction in the point cloud that most closely aligns with the geometric structure of the stem, thereby approximating the central axis of the stem.

The separated stem point cloud is input into the PCA model, and the principal direction vector is extracted through the feature vector corresponding to the maximum eigenvalue as the estimated result of the main axis of the maize stem. The acquisition of this main axis provides a stable and geometrically meaningful reference benchmark for subsequent point cloud slicing, region segmentation, and structural feature analysis.

Assume that the processed maize stem point cloud consists of N three-dimensional points, denoted as ${\left\{p_{i}={\left(x_{i},y_{i},z_{i}\right)}^{\tau }\right\}}_{i=1}^{N}$. To simplify subsequent analysis, the three-dimensional coordinates $p_{i}$ of all points are organized into a matrix $X\in R^{N\times 3}$, and then the point cloud undergoes PCA dimension reduction processing.

The PCA method extracts the principal directions of the data by calculating the covariance matrix of the point cloud data (Salih Hasan and Abdulazeez, 2021). Specifically, the covariance matrix C is: $C=\frac{1}{N}\sum_{i=1}^{N}\left(p_{i}-\bar{p}\right){\left(p_{i}-\bar{p}\right)}^{\tau }$, where $\bar{p}$ is the mean vector of the point cloud. By performing an eigenvalue decomposition of the covariance matrix, we obtain the eigenvectors $v_{1}$, $v_{2}$ and $v_{3}$, where $v_{1}$ represents the principal axis direction, which corresponds to the primary growth direction of the stem. The principal axis direction is calculated using Equation 8:

(8) $$v_{1}=\arg\underset{v}{max}\left(v^{\tau }Cv\right)$$

Step 2: Node detection based on structural variation.

Maize stems show clear radial swelling and curvature changes at node regions, whereas internodes maintain relatively stable geometric morphology. Based on this biological characteristic, the stem point cloud is uniformly partitioned into 15 slices along the principal axis to ensure sufficient structural resolution. For each slice, cross-sectional analysis is conducted by projecting the local point cloud onto the plane perpendicular to the stem axis (XZ-plane), followed by ellipse fitting to extract geometric properties.

The major axis of the fitted ellipse quantifies radial expansion and thus reflects stem swelling, while the minor axis represents secondary diameter variation. By analyzing the sequential trend of major axis values across slices, node regions can be identified as those exhibiting a distinctive “increase–peak–decrease” pattern. Following the anatomical order from ground level, the first and second detected peaks correspond to the first and second nodes, respectively. Consequently, the point cloud region between these peaks is automatically defined as the second internode ROI.

**Figure 5:**
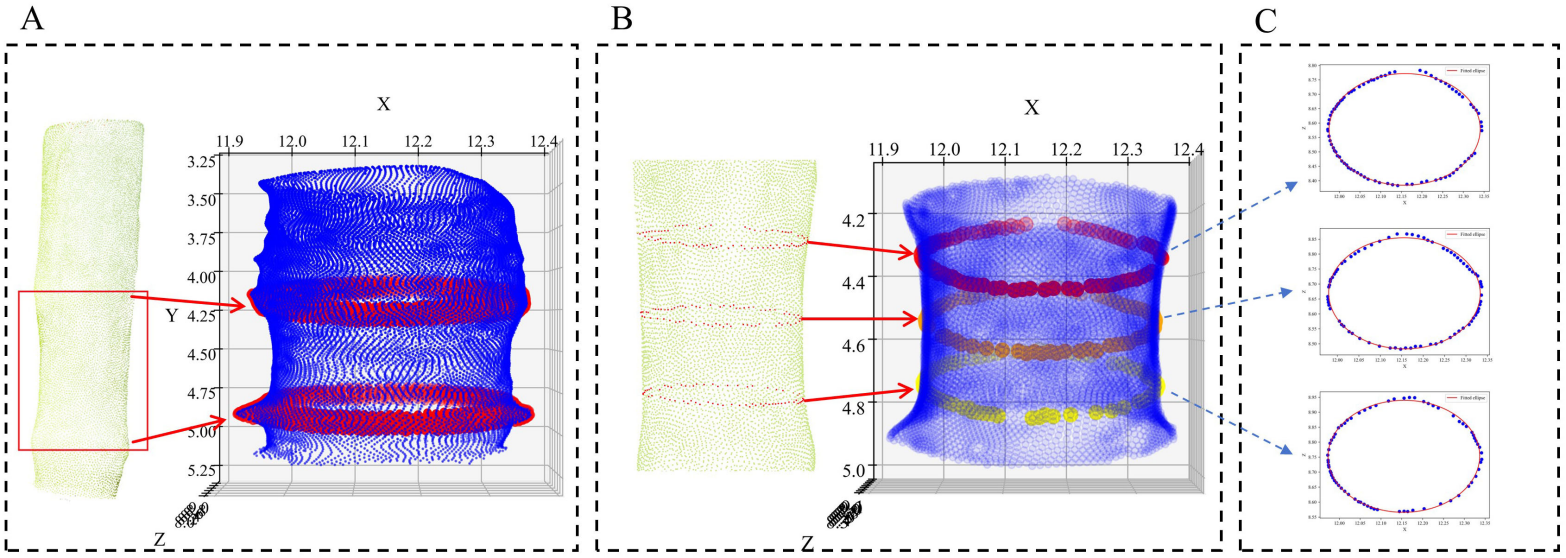
Stem diameter measurement. **(A)** Region of interest extraction. **(B)** Extraction of three cross sections of the region of interest. **(C)** Ellipse fitting results.

Furthermore, the slice with the most pronounced turning point from expansion to contraction is highlighted in red in the 3D view, as illustrated in **Figure 5A**, indicating the key boundary of the internode swelling region.

By locating the area between the two swollen areas, we can determine the area of interest on the maize stem. This method can automatically identify the swollen area on the stem starting from the second node and provide accurate area localization for subsequent stem diameter measurement.

Step 3: ROI extraction and stem diameter measurement.

After automatically identifying the second internode region based on structural variation, three cross-sectional slices are extracted perpendicular to the principal axis within this ROI. The slices are evenly spaced along the axis to ensure uniform sampling of the internode region. This automated selection strategy ensures that the slices correspond closely to the positions typically used in manual measurements, maintaining anatomical consistency and allowing meaningful comparison.

For each slice, the local point cloud is projected onto the XZ plane, and ellipse fitting is applied to estimate the major and minor axes, corresponding to the primary and lateral stem diameters, respectively. in **Figure 5B** shows the visualization results extracted from three cross sections of the region of interest, while in **Figure 5C** shows the cross sections after elliptical fitting.This procedure allows fully automatic, reproducible, and anatomically meaningful stem diameter measurement without manual intervention.

**Figure 6:**
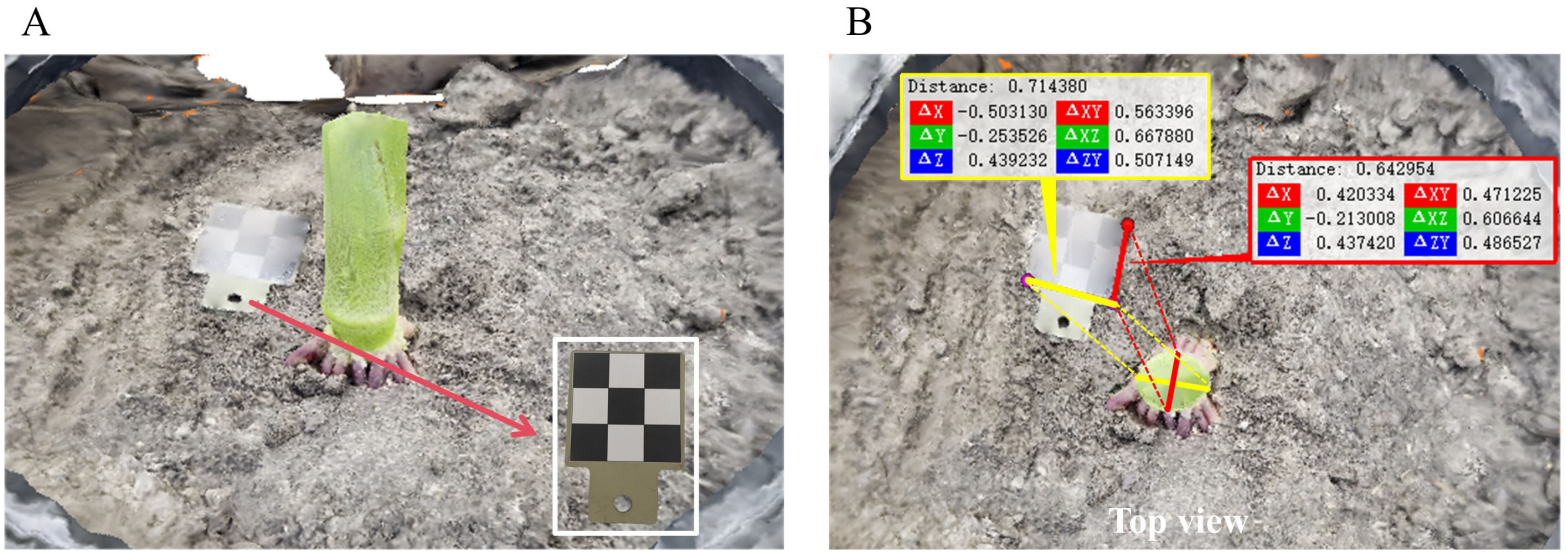
Checkboard scale reference and distance selection diagram. **(A)** Experimental image of checkboard as scale reference. **(B)** Top view checkboard distance selection.

Since multi-view reconstruction relies on the estimation of the relative position between images, there is a scale deviation between the reconstructed point cloud model and the actual physical scene. In order to achieve true size restoration, this paper introduces a 3×3 checkerboard calibration plate with a side length of 15 mm as a scale reference during data collection. The overall length and width of the checkerboard is 45 mm, and it is placed near the stem to ensure that it is visible in the multi-view images, as shown in in **Figure 6A**. The checkerboard calibration plate in the reconstructed 3D point cloud was measured using CloudCompare, and the distances of the checkerboard calibration plate parallel to the major axis and minor axis of the maize stem were taken, as shown in in **Figure 6B**. Subsequently, the scale transformation formula, as shown in Equation 9, was used to obtain the stem diameter measurement results reflecting the actual physical dimensions.

(9) $$\frac{S}{L}=\frac{45}{Q}$$

Among them, S represents the actual stem diameter, L represents the length of the major or minor axis of the ellipse, and Q represents the relative distance between the edges of the checkerboard in the three-dimensional dense point cloud.

### 2.3 Experimental setup

The processed 72 sets of maize stem point cloud data were divided into 50 training sets, 11 validation sets, and 11 test sets. The statistical data corresponding to the point cloud annotations of the dataset are shown in **Table 3**. All experiments were conducted on a single NVIDIA GeForce RTX 4070 Ti SUPER GPU using the PyTorch library on the PyCharm platform. The model was trained using the AdamW optimizer for 32 epochs starting from scratch. The learning rate was set to 0.001. During model training, the model parameters corresponding to the epoch with the highest IoU on the validation set were saved for performance testing.

**Table 3:** Statistics of the dataset.

Dataset	Number of samples	Number of background points	Number of stem points
Training	50	19195306	860033
Validation	11	3691526	192477
Test	11	4187111	211921

### 2.4 Evaluation indicators

#### 2.4.1 Model evaluation indicators

To conduct a comprehensive and precise quantitative assessment of the performance of the aforementioned algorithms, this study selected Mean Accuracy (mAcc) and Mean Intersection over Union (mIoU) as core evaluation metrics. Mean Accuracy (mAcc) is calculated by first determining the accuracy rate for each category and then taking the average, thereby further reducing the excessive influence of the background category on the overall evaluation results. This indicator reflects the performance of the model in different categories from a more comprehensive perspective, allowing us to evaluate the model’s performance in a more three-dimensional and complete manner. Mean Intersection over Union (mIoU) comprehensively considers multiple key factors such as true positives, false positives, and false negatives. In tasks such as image segmentation, it can measure the performance of the model in all aspects without blind spots, providing strong support for accurately evaluating the segmentation capabilities of the model in complex data environments. The formulas for the two evaluation metrics are shown in Equations 10a, Equations 10b, Equations 11a, and Equations 11b, respectively, laying a solid theoretical foundation for subsequent quantitative analysis. Where N represents the total number of classes; $TP_{i}$ denotes the number of points correctly predicted as belonging to category i (True Positive); $FP_{i}$ denotes the number of points incorrectly predicted as belonging to category i (False Positive); $FN_{i}$ denotes the number of points that should have been predicted as belonging to category i but were not correctly identified (False Negative).

Mean Accuracy (mAcc) formula:

(10a) $$Acc_{i}=\frac{TP_{i}}{TP_{i}+FN_{i}}$$

(10b) $$mAcc=\frac{1}{N}\sum_{i=1}^{N}Acc_{i}=\frac{1}{N}\sum_{i=1}^{N}\frac{TP_{i}}{TP_{i}+FN_{i}}$$

The formula for calculating the mean intersection over union (mIoU) is:

(11a) $$IoU_{i}=\frac{TP_{i}}{TP_{i}+FP_{i}+FN_{i}}$$

(11b) $$mIoU=\frac{1}{N}\sum_{i=1}^{N}IoU_{i}=\frac{1}{N}\sum_{i=1}^{N}\frac{TP_{i}}{TP_{i}+FP_{i}+FN_{i}}$$

#### 2.4.2 Maize stem diameter evaluation index

To verify the accuracy of the three-dimensional measurement method for maize stem diameter in the field combining RLRSA-PointNet++ and structural feature fitting, this paper compares and analyzes the manually measured values (obtained using a vernier caliper) with the measurement results based on three-dimensional point clouds. The primary metrics used to assess measurement accuracy include the Mean Absolute Percentage Error (MAPE), Mean Absolute Error (MAE), Root Mean Square Error (RMSE), and Coefficient of Determination (R²) (Chicco et al., 2021). The formulas for these metrics are shown in Equations 12, Equations 13, Equations 14, and Equations 15.Here, n represents the sample size; $y_{i}$ denotes the manually measured value for the i-th sample (stem diameter measured using a vernier caliper); ${\hat{y}}_{i}$ represents the predicted value for the i-th sample (stem diameter measured using the 3D point cloud method); and $\bar{y}$ denotes the average of the manually measured values.

(12) $$MAPE=\frac{1}{n}\sum_{i=1}^{n}\frac{\left|{\hat{y}}_{i}-y_{i}\right|}{k_{i}}\times 100\%$$

(13) $$MAE=\frac{1}{n}\sum_{i=1}^{n}\left|{\hat{y}}_{i}-y_{i}\right|$$

(14) $$RMSE=\;\sqrt{\frac{1}{n}\sum_{i=1}^{n}{\left({\hat{y}}_{i}-y_{i}\right)}^{2}}$$

(15) $$R^{2}=1-\frac{\sum_{i=1}^{n}{\left(y_{i}-{\hat{y}}_{i}\right)}^{2}}{\sum_{i=1}^{n}{\left(y_{i}-\bar{y}\right)}^{2}}$$

Building upon the evaluation of error metrics, this study further incorporates residual analysis, as well as confidence interval (CI) and prediction interval (PI) analyses. Residual analysis is employed to assess the distribution characteristics of deviations between the predicted values and the ground truth, as expressed in Equation 16. The CI is used to quantify the credible range of the regression curve in estimating the population mean, as given in Equation 17. In contrast, the PI reflects the potential fluctuation range of new sample points under specific independent variable conditions, as formulated in Equation 18. Here, $x_{0}$ denotes the ground truth of maize stem diameter obtained through manual measurement, while $\hat{y}\left(x_{0}\right)$ represents the predicted stem diameter value.The corresponding equations are as follows:

Residual analysis formula:

(16) $$e_{i}=y_{i}-{\hat{y}}_{i}$$

Confidence Interval (CI) of regression fitting:

(17) $$CI:\hat{y}\left(x_{0}\right)\pm t_{\frac{\alpha }{2},n-2}\cdot s\cdot \sqrt{\frac{1}{n}+\frac{{\left(x_{0}-\bar{x}\right)}^{2}}{\sum_{i=1}^{n}{\left(x_{i}-\bar{x}\right)}^{2}}}$$

Prediction Interval (PI) of regression fitting:

(18) $$PI:\hat{y}\left(x_{0}\right)\pm t_{\frac{\alpha }{2},n-2}\cdot s\cdot \sqrt{1+\frac{1}{n}+\frac{{\left(x_{0}-\bar{x}\right)}^{2}}{\sum_{i=1}^{n}{\left(x_{i}-\bar{x}\right)}^{2}}}$$

To further verify whether statistically significant differences exist between the predicted values obtained from the method and the actual values, we employed a paired samples t-test. For each sample group, the difference was calculated as the actual measurement minus the predicted value, yielding the mean $\bar{d}$ and standard deviation ${\text{s}}_{d}$ of the differences. The formula for the t-test statistic is shown in Equation 19:

(19) $$t=\frac{\bar{d}}{{\text{s}}_{d}/\sqrt{n}}$$

where n is the sample size. The null hypothesis states that there is no significant difference between the means of the two groups. When the p-value is greater than 0.05, it indicates no statistically significant difference between the predicted results and the actual measured results, demonstrating that the model possesses high measurement consistency.

## 3 Results

### 3.1 Semantic segmentation method results and comparison

There is a certain degree of class imbalance in the experimental data, and this distribution difference may affect the performance evaluation of semantic segmentation models, especially when using metrics such as overall accuracy, which can mask the model’s ability to recognize minority classes.

To comprehensively evaluate the performance of different models in handling semantic segmentation tasks with imbalanced category distributions in point clouds, this paper systematically compares DGCNN (Phan et al., 2018), PointNet (Charles et al., 2017), PointNet++, and the RLRSA-PointNet++ model proposed in this paper from three dimensions: mean loss (mloss), mean accuracy (mAcc), and mean intersection over union (mIoU). The specific results are shown in **Table 4**.

**Table 4:** Comparison of experimental results for the four algorithms.

Model	mloss	mAcc	mIOU
DGCNN	1.102	0.891	0.902
PointNet	0.723	0.942	0.922
PointNet++	0.764	0.934	0.934
RLRSA-PointNet++	**0.499**	**0.943**	**0.980**

Bold values indicate the best performance of the results.

The experimental results show that the RLRSA-PointNet++ model achieves the best performance in all evaluation metrics. Its average loss value is 0.499, which is significantly better than PointNet (0.723), PointNet++ (0.764), and DGCNN (1.102), indicating that the model converges more easily and is more stable during training. In terms of average accuracy, the RLRSA-PointNet++ model achieved 0.943, outperforming PointNet++ (0.934) and DGCNN (0.891), and showing a slight improvement compared to PointNet (0.942). Additionally, the RLRSA-PointNet++ model achieved an optimal average intersection-over-union ratio of 0.980, showing a significant improvement over PointNet++ (0.934), PointNet (0.922), and DGCNN (0.902), fully demonstrating its advantages in category discrimination capabilities. In summary, the RLRSA-PointNet++ model exhibits more stable training convergence and higher overall prediction accuracy, reflecting its powerful feature expression and discrimination capabilities in handling category imbalance and complex structure point cloud data, effectively supporting the fine segmentation task of maize stem thick areas.

**Figure 7:**
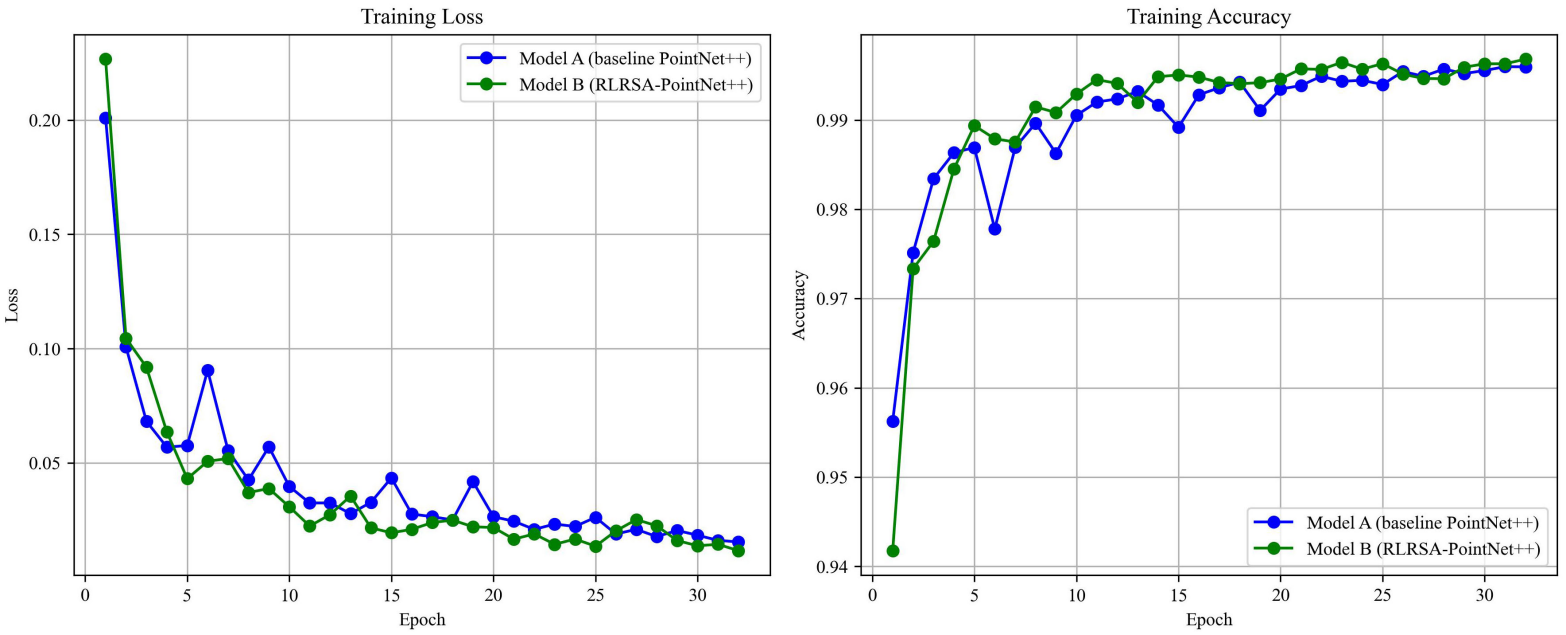
Training set loss and accuracy change curve.

The loss and accuracy change curves of the improved PointNet++ and the original PointNet++ training set are shown in **Figure 7**. The RLRSA-PointNet++ model demonstrated better convergence speed and lower training loss during training, while also achieving more stable and higher accuracy results, indicating that the model has advantages in feature extraction and classification discrimination, thereby improving training efficiency and model performance. Compared to the traditional PointNet++, the improved PointNet++ effectively enhances the model’s expressive capability and robustness.

**Figure 8:**
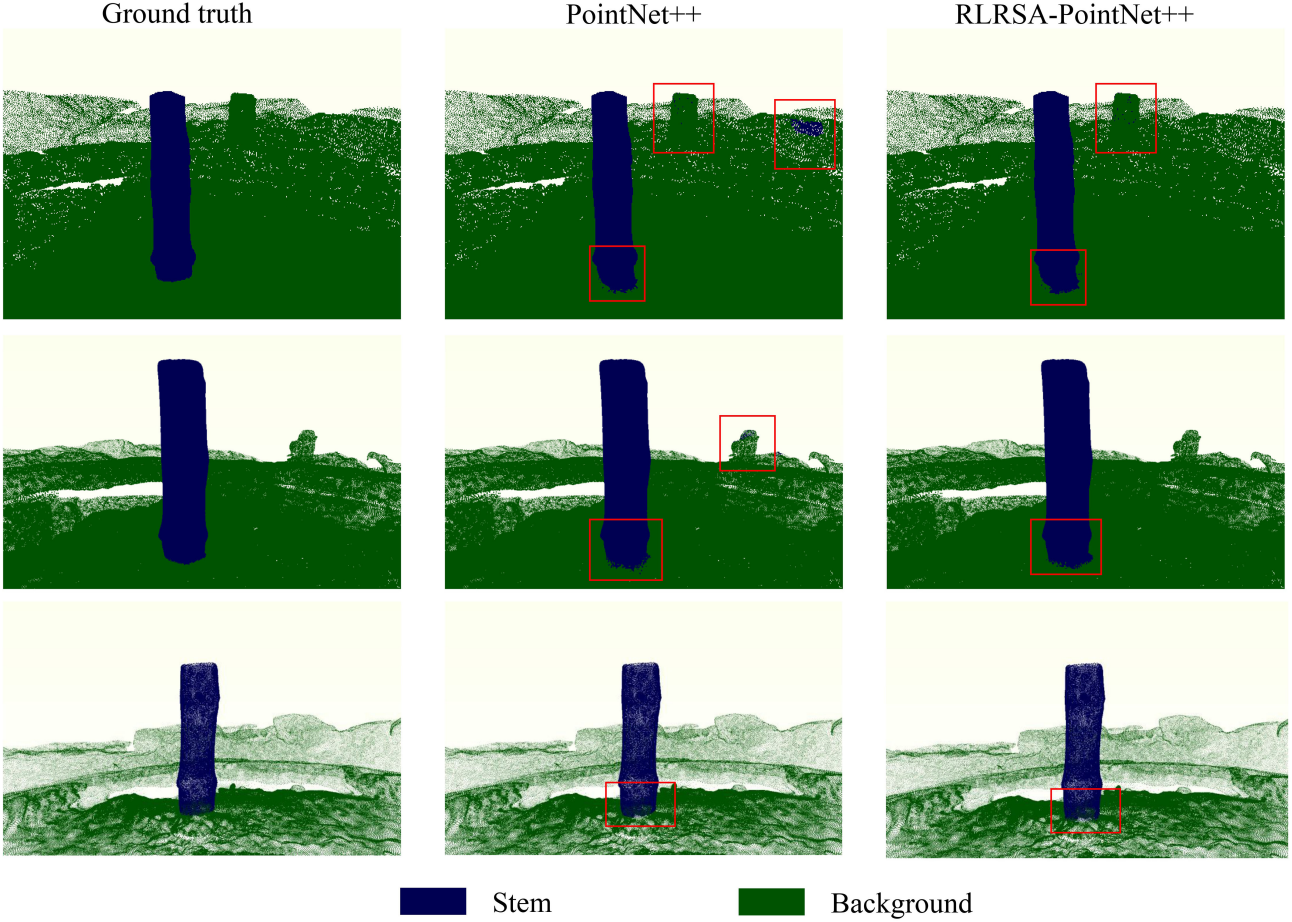
Comparison of segmentation results between the original dataset annotation and PointNet++ and RLRSA-PointNet++.

The corresponding data segmentation results are shown in **Figure 8**. As can be seen from the figure, the improved PointNet++ can better segment the stems and the background. At the junction between the stems and the ground, as well as in the background, the segmentation results of similar maize stems are significantly better than those of the original PointNet++.

The area shown in the red box in **Figure 8** mainly reflects the model’s ability to segment target stems and ground as well as non-target objects. Although these areas are not directly relevant to stem diameter measurement, misclassification can seriously interfere with subsequent morphological parameter calculations, especially in the process of extracting the main axis direction of the stem using principal component analysis (PCA). If background points are misclassified as stems, it will cause the main axis estimation of the point cloud to shift, thereby affecting the accuracy of cross-section extraction and stem diameter measurement along the major and minor axes.

In contrast, the RLRSA-PointNet++ model exhibits higher segmentation accuracy in these critical boundary and background areas, effectively reducing misidentification and significantly improving the stability and measurement accuracy of the principal axis extraction. Therefore, although the red box area in the figure is not a critical structure, its improved performance has important practical significance for the entire morphological measurement process.

### 3.2 Ablation experiment

In order to systematically verify the contribution of each key module in the improved PointNet++ model proposed in this paper to the two-classification task of maize stems and background point clouds, ablation experiments were designed for the Relative Position Encoding (RPE), local rearrangement module (LGRM), and Local Region Self-Attention mechanism (LRSA). By gradually removing or combining these modules, the performance of different model structures was compared on multiple evaluation indicators, and the independent effects and synergistic effects of each module were analyzed.

**Figure 9:**
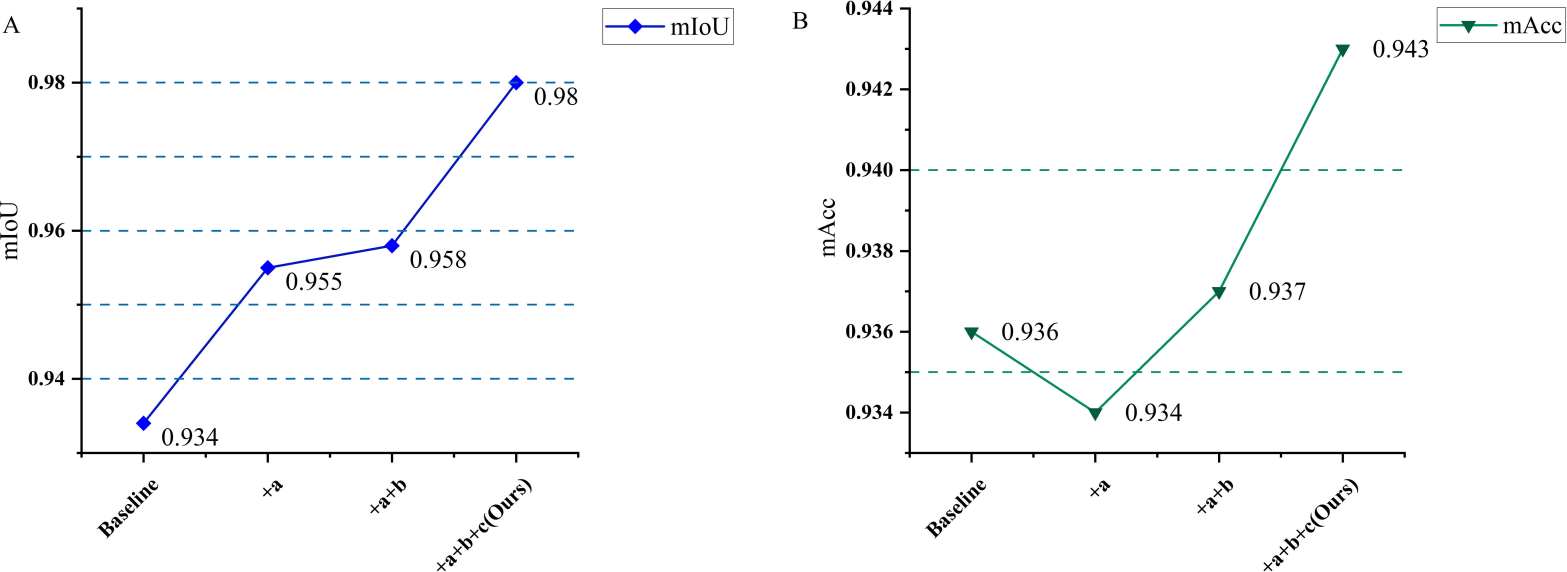
Experimental results of adding modules (a) RPE; (b) LGRM; (c) LRSA. **(A)** mIoU results of the ablation experiments. **(B)** mAcc results of the ablation experiments.

Six model groups were specifically designed for comparison: (A) the original PointNet++ baseline model; (B) the baseline model with the RPE module added; (C) the baseline model combined with the LGRM module; (D) the baseline model combined with the RPE and LGRM modules; (E) the baseline model combined with the RPE and LRSA modules; (F) an improved version of the baseline model that fully integrates the RPE, LGRM, and LRSA modules. All models use the same dataset, training strategy, and parameter configuration to ensure the fairness and comparability of the experiments. The experimental results are shown in **Table 5**, and the experimental results of gradually adding modules are shown in **Figure 9**.

**Table 5:** Ablation experiment results.

Number	Model structure	mIoU	mAcc
A	Original PointNet++ baseline model	0.934	0.936
B	baseline+RPE	0.955	0.934
C	baseline+LGRM	0.958	0.942
D	baseline +RPE+LGRM	0.958	0.937
E	baseline +RPE+LRSA	0.952	0.935
F	baseline+RPE+LGRM+LRSA (Our model)	**0.980**	**0.943**

Bold values indicate the best performance of the results.

From the analysis of the experimental results, it can be seen that the original PointNet++ model (A) achieved benchmark performance of mIoU 0.934 and mAcc 0.936 without introducing any improvement modules. After introducing the RPE module (B), mIoU improved to 0.955, indicating that relative position encoding helps enhance the model’s ability to represent local geometric relationships in point clouds. However, mAcc slightly decreased, possibly due to overfitting of the model on certain categories, leading to slight fluctuations in overall accuracy. Further introduction of the LGRM module (C) enabled the model to maintain a high mIoU (0.958) while improving mAcc to 0.942, validating the optimization effect of local structure rearrangement on feature representation within neighborhoods. When RPE and LGRM were introduced simultaneously (D), mIoU did not further improve, but mAcc slightly decreased to 0.937, indicating potential functional overlap or interference between the two. In contrast, when RPE is combined with LRSA (E), the model achieves mIoU 0.952 and mAcc 0.935, indicating that LRSA has certain advantages in modeling long- and short-range dependencies, but its gains are insufficient when not combined with local structure optimization. Finally, the complete model integrating all three modules (F) achieves the highest mIoU (0.980) and mAcc (0.943), showing a significant improvement over the baseline model. This indicates that RPE provides effective positional priors, LGRM improves local structure representation, and LRSA helps capture global contextual information. The synergistic interaction of these three components significantly optimizes the network’s accuracy and stability. In summary, ablation experiments thoroughly validate the effectiveness and necessity of the proposed improved modules in enhancing point cloud semantic segmentation performance.

### 3.3 Stem diameter measurement results and analysis

**Figure 10:**
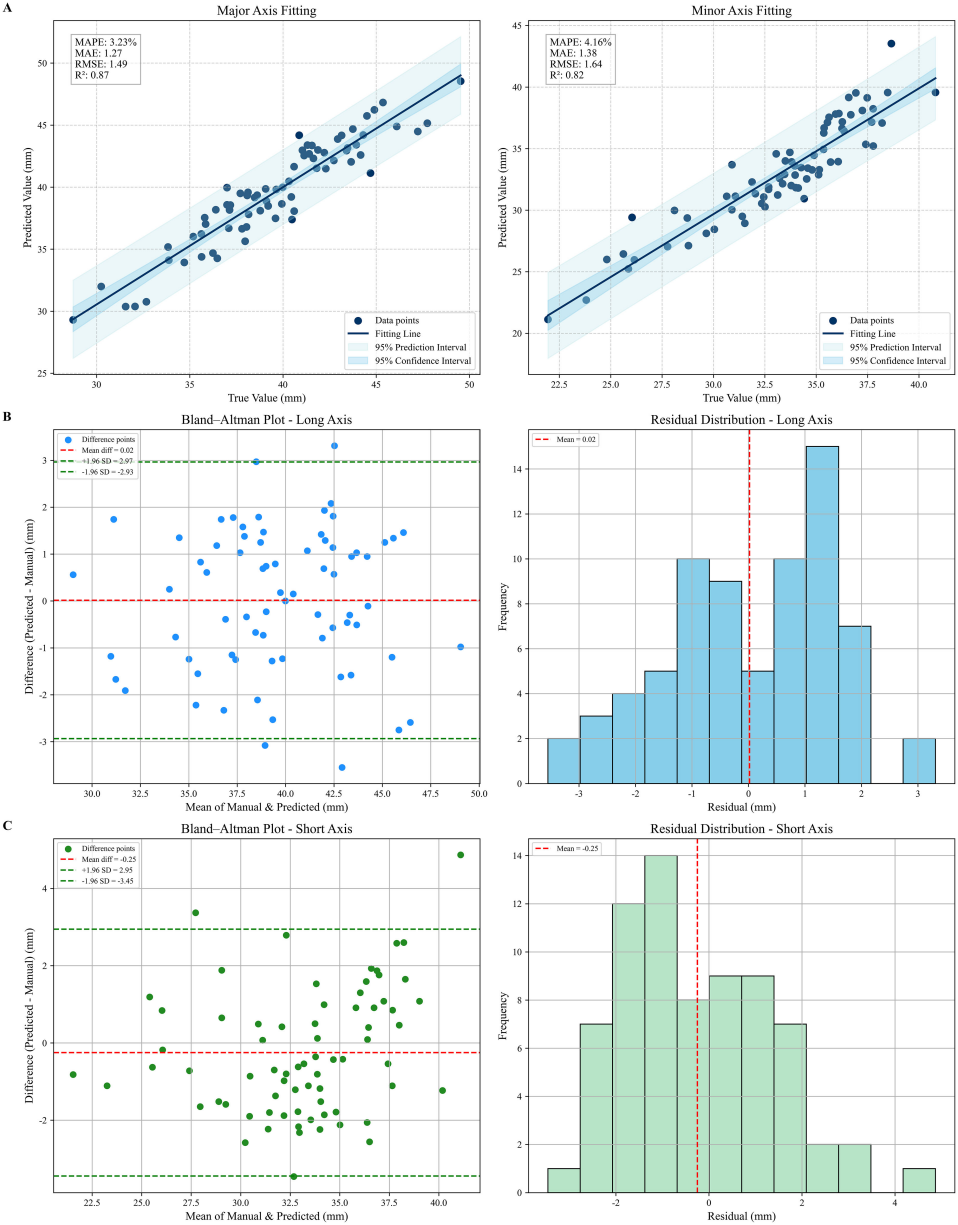
Linear fitting and residual analysis of maize stem diameters (major and minor axes). **(A)** Linear fitting results. **(B)** Residual analysis of the major axis diameter of maize stems. **(C)** Residual analysis of the minor axis diameter of maize stems.

This study used 72 sets of three-dimensional dense point cloud data of maize stems as experimental material and conducted an error analysis of the stem diameter measurements obtained using the RLRSA-PointNet++ point cloud semantic segmentation algorithm and structural feature fitting method and the manual measurements obtained using a vernier caliper. The MAPE of the major axis stem diameter of the 72 groups of maize was 3.23%, the MAE was 1.27 mm, the RMSE was 1.49 mm, and the R^2^ was 0.87. The MAPE of the minor axis stem diameter of the 72 groups of maize was 4.16%, the MAE was 1.38 mm, the RMSE was 1.64 mm, and the R^2^ was 0.82. In order to intuitively show the differences between the major axis stem diameter and minor axis stem diameter measurement results based on the improved PointNet++ and the manual measurement results, this study performed linear fitting on these data, and the fitting results are shown in **Figure 10A**.

In the figure, it can be observed that the 95% confidence interval (CI) of the stem major axis fitting results is relatively narrow and more convergent, with a width significantly smaller than that of the prediction interval (PI). This indicates that the model provides stable estimates of the regression coefficients along the major axis, and the overall fitting relationship demonstrates high reliability. Furthermore, most test points fall within the PI band, suggesting that the model exhibits strong single-point predictive capability in the major axis direction, with only a few outliers contributing to error fluctuations.

By contrast, the stem minor axis fitting results exhibit both wider CI and PI, particularly in higher-value regions where the prediction band expands considerably. This reflects greater uncertainty in parameter estimation for the minor axis, with relatively weaker stability in single-point predictions.

In summary, the predictive performance along the major axis surpasses that of the minor axis: the regression relationship is more robust and the prediction results more reliable. However, predictions of the minor axis exhibit certain limitations, which necessitate further feature optimization or an increase in sample size to enhance accuracy.

In the measurement of major axis stem diameter, the residual range is primarily concentrated within ±2 mm, with the error distribution generally following a symmetric distribution and no obvious skewness. This indicates that the prediction errors exhibit strong randomness and no systematic overestimation or underestimation trends. Most residuals are concentrated around 0, suggesting that the model possesses high fitting accuracy and good generalization capability (McInerney et al., 2017).

In minor axis stem diameter measurements, the residuals are slightly larger, with some samples exhibiting prediction errors exceeding ±2 mm. However, the overall distribution remains within an acceptable range, indicating that the model’s fitting capability in the minor axis direction is slightly lower than in the major axis direction but still achieves good predictive performance. Some larger residuals may stem from lower point cloud density in the minor axis region, blurred edge contours, or uncertainties in fitting the major and minor axes of the ellipse.

The results of the paired sample t-test indicate that for maize stem diameter along the major axis, t = -0.0932, p = 0.9260; and for maize stem diameter along the minor axis, t = 1.2995, p = 0.1980. This demonstrates that there is no statistically significant difference (p > 0.05) between the actual measured values and the algorithm-predicted values for both the major and minor axes. The t-value for the major axis is close to zero, indicating minimal error. Although the t-value for the minor axis is slightly larger, it remains within a reasonable margin of error. These results demonstrate high consistency between the model predictions and actual values, with no apparent systematic bias. Therefore, this method exhibits high reliability and practicality for measuring maize stem diameter.

To further evaluate the reliability of the proposed stem diameter estimation method, Bland–Altman analysis and residual distribution assessment were conducted using manual measurements as field data. As shown in **Figure 10B** and **Figure 10C**, the mean difference between estimated major axis diameters and reference major axis diameters was 0.02 mm, approaching zero, indicating no systematic bias. The 95% consistency limits ranged from −2.93 mm to 2.97 mm, with over 93% of observations falling within this interval, demonstrating high consistency.

Similarly, the mean deviation for minor axis diameter was −0.25 mm, with 95% consistency limits ranging from −3.45 mm to 2.95 mm. This indicates a slight underestimation, yet the stability is good and falls within an acceptable range. The residual histogram further confirms that most errors for both major and minor axis diameters cluster within ±2 mm, with no discernible heavy tails or bimodal distributions. Peak error values predominantly occur between −1 mm and 1.5 mm, reflecting the model’s high precision and robust stability.

Moreover, no discernible trend of increasing residuals with larger stem diameter values was observed, indicating negligible heteroscedasticity and proportional bias. These results confirm that point cloud-based corn stem diameter measurements achieve consistent alignment with manual measurements, exhibiting minimal numerical deviation and narrow error dispersion, thereby delivering stable and consistent predictive performance.

In summary, the improved PointNet++-based 3D measurement method for maize stem diameter shows good fitting accuracy and small prediction errors in both the major and minor axis directions. Related studies show that for small-sized crop phenotype parameters, measurement errors within 20% can be considered acceptable and have good practical value (Jin et al., 2022).

## 4 Discussion

### 4.1 Error distribution and failure-case analysis

To complement the overall evaluation metrics (MAE and RMSE), we further analyzed the error distribution and representative failure cases to gain insights into the robustness and limitations of the proposed stem diameter measurement method under real-world field conditions. the majority of estimation errors were concentrated within ±1 mm, demonstrating high measurement consistency. However, a small subset of cases exhibited larger deviations, with a maximum deviation of 2–3 mm observed in the test set.

These outliers were primarily associated with three scenarios:

1. Partial occlusion or missing point cloud regions: Portions of the stem surface may be absent due to camera blind spots or leaf occlusion, causing the fitted ellipse to be biased toward the preserved region and resulting in underestimation of the stem diameter.
2. Sparse or locally noisy point cloud regions: Areas with low point density or surface irregularities lead to unstable ellipse fitting, inducing local fluctuations in the estimated major axis length.
3. Irregular or curved stem morphology: Stems with abnormal bending or non-circular cross sections violate the near-elliptical assumption, producing over- or under-estimated diameters.

These observations highlight that the accuracy of ellipse-based diameter measurement is closely linked to both the structural regularity of the stem and the completeness of 3D reconstruction.

During actual field deployment, factors such as the standardization of data collection processes, the quality of 3D point cloud reconstruction, and environmental conditions must also be considered.In real field environments with severe occlusions, motion blur, or lighting variations, segmentation stability may be compromised. Furthermore, the method assumes relatively regular stem structures by default, leaving room for improvement in adaptability to curved or irregular samples. Future work will explore incorporating sensor point clouds such as LiDAR or ToF, and investigate network architectures that either bypass reconstruction or incorporate occlusion handling capabilities to further enhance practical field deployment.

### 4.2 Analysis of the impact of class imbalance on model performance

In this study, the collected point cloud data exhibited significant class imbalance, with over 300,000 points in the background class and only approximately 20,000 points in the stem class. This imbalanced distribution posed numerous challenges during model training and performance evaluation. Since background-class point clouds dominate, relying solely on overall accuracy as an evaluation metric often leads to results biased toward the numerically dominant category, making it difficult to fully reflect the model’s ability to identify minority classes (such as stems), thereby affecting the segmentation performance of critical regions.

Additionally, category imbalance may cause category bias during the training phase, especially for categories with fewer samples, where the model struggles to learn representative feature expressions, leading to decreased segmentation performance, particularly in stem regions with complex boundaries and rich structural details (Thabtah et al., 2020). In our study, we selected PointNet, PointNet++, and DGCNN as the primary comparative baselines because they represent the most classical and widely adopted point-based architectures, and serve as strong foundational models for evaluating improvements in local geometric feature learning and structural context aggregation. Experimental results show that while traditional point cloud segmentation models like DGCNN and PointNet++ perform reasonably well in terms of overall accuracy, they lag significantly behind the RLRSA-PointNet++ model proposed in this paper in terms of the intersection-over-union (IoU) metric for minority class recognition quality. The latter effectively enhances sensitivity and discriminative ability toward minority class features, achieving more precise category differentiation.

Despite the emergence of newer 3D point cloud segmentation models such as KPConv (Thomas et al., 2019) and PointNext (Qian et al., 2022), these methods were not included in the comparative experiments mainly due to their substantially higher computational costs, complex hyperparameter configurations, and incompatibility with our specific agricultural point cloud characteristics, such as non-uniform density, elongated geometry, and node-like structural patterns. In particular, the uneven density distribution of the point cloud studied in this paper led to situations where high accuracy was achieved in actual experiments, yet segmentation results were extremely poor. Both models tended to classify stem sections as background areas, preventing accurate stem segmentation. Consequently, subsequent stem diameter acquisition experiments could not proceed.

In our study, to mitigate the impact of class imbalance, relative position encoding and a local rearrangement module were employed to enhance the local structural representation and discriminative ability of minority point cloud classes, thereby improving the segmentation performance of maize stems to some extent. However, due to the scarcity and structural complexity of minority class samples, this improvement remains limited, and further optimization and refinement are urgently needed in future research.

In summary, class imbalance is not only a data-level problem, but also an important factor that constrains the improvement of model feature learning and discrimination capabilities. In the complex field environment of maize stems, the scarcity and structural complexity of minority class samples further increase the difficulty of model training and application. Future research should focus more on designing adaptive and efficient class balancing mechanisms, combining multi-source data fusion, dynamic weight adjustment, and deep feature enhancement techniques to improve model performance in complex imbalanced point cloud scenarios, thereby promoting the widespread application of point cloud semantic segmentation technology in agricultural precision detection.

### 4.3 Segmentation performance improvement and structural parameter extraction method evaluation

The RLRSA-PointNet++ model proposed in this paper is based on the classic PointNet++ framework and innovatively introduces Relative Position Encoding, local reordering modules, and Local Region Self-Attention mechanisms, effectively enhancing the model’s ability to express local geometric details in unstructured point clouds. Compared to traditional methods, this design not only improves the model’s ability to perceive complex spatial structures but also demonstrates higher segmentation stability and accuracy in complex interference regions such as stems, backgrounds, and ground surfaces, overcoming the limitations of the original PointNet++ in spatial structure modeling. Relative position encoding explicitly captures the spatial relationships between points and their neighborhoods, promoting the precise aggregation of local features. The local reordering module optimizes the self-attention mechanism’s capture of local structures by adjusting the order of neighboring points. Additionally, the Local Region Self-Attention mechanism further strengthens the model’s adaptive weight allocation for local important features, improving its ability to identify fine-grained structures.

We compare our method with recent Transformer-based approaches specifically developed for plant point cloud segmentation. Among these, Plant Segmentation Transformer (PST) (Du et al., 2023)proposes a dynamic voxel feature encoder, dual-window self-attention module, and dense feature propagation mechanism. They applied it to rapeseed point cloud data, achieving promising semantic segmentation performance. However, PST and related studies typically rely on voxelization or hybrid structured representations to enable Transformer attention mechanisms on regularized features. In contrast, our approach operates directly on raw, unstructured 3D point cloud data, focusing primarily on enhancing local geometric grouping and lightweight regional attention within the PointNet++ backbone. This design preserves the geometric fidelity of slender cylindrical structures like maize stems, better retaining fine local boundaries between stem and background. Consequently, our approach is particularly suited for stem-background segmentation tasks requiring local topology preservation and avoiding voxelization.

Nevertheless, this method still has certain limitations, such as the fact that the effects of local reordering and self-attention mechanisms may be limited in cases of extremely low point cloud density or severe stem occlusion. Future research will further explore multi-view fusion and temporal information integration to enhance the model’s robustness and generalization capabilities.

In terms of stem diameter extraction, the elliptical structure feature fitting strategy based on slices proposed in this paper can efficiently extract the major and minor axis parameters of the cross-section for low-density, non-uniformly distributed stem point clouds in the field. This method captures the key geometric features of the stem cross-section by fitting ellipses to multi-level slices. Experiments demonstrate its good adaptability and robustness under different growth states and posture changes, providing a solid data foundation for precise modeling of plant structure and phenotypic trait analysis in complex agricultural environments.

However, when comparing the results with manual measurements, it is important to note that the positions of internodes selected for manual measurements may be subject to some degree of experience and subjectivity, while the positions of slices extracted by the algorithm are automatically determined based on point cloud density and spatial distribution characteristics. As a result, there may be minor discrepancies in specific positions between the two methods. Although this study employs a multi-point averaging strategy to minimize the impact of such discrepancies, the differences may still affect the consistency of the comparison to some extent. Future work will further optimize the method from the perspectives of consistency in slice positions and precise alignment of structural regions to enhance the overall accuracy and reproducibility of the analysis.

### 4.4 Computational efficiency and throughput potential

Beyond segmentation accuracy, computational efficiency is also a critical consideration for practical deployment in large-scale phenotyping scenarios. To evaluate this aspect, we analyzed the runtime costs across the entire workflow. Our experimental setup utilized an Intel Core i7-13700K CPU and a 16GB RTX 4070 Ti SUPER GPU.

Reconstructing a single maize stem using MVS requires 90 images at a resolution of 3840×2160. Data acquisition and 3D point cloud reconstruction take approximately 5–6 minutes. This demonstrates that reconstruction using high-resolution images places significant demands on computational power. Training the deep learning model from scratch requires 32 epochs, with each epoch taking approximately 10–12 minutes, resulting in a total training time of about 5–6 hours. Since model training is an offline, one-time process, this cost does not constrain subsequent large-scale deployment. The average processing time for a single maize plant is approximately 2 to 5 seconds, encompassing both point cloud segmentation and stem diameter calculation.

The data above demonstrates that this inference efficiency holds significant potential for high-throughput applications. Although currently implemented in offline mode, the relatively low inference time indicates that real-time or near-real-time deployment could be achieved through further model optimization, adoption of lightweight implementations, or specialized hardware acceleration.

### 4.5 Limitations and future improvements

Despite the promising performance of the proposed RLRSA-PointNet++ framework and slice-based elliptical fitting strategy, several limitations remain regarding its deployment in real-world agricultural scenarios. First, the method relies heavily on the quality, completeness, and structural consistency of point clouds, which, although collected directly in field conditions, are still susceptible to challenges such as leaf occlusion, wind-induced plant motion, sunlight variation, soil interference, and uneven camera viewpoints. These real-world factors may lead to sparse or locally distorted point cloud regions, which negatively influence both semantic segmentation and stem diameter estimation. While our point clouds were acquired under actual farmland conditions, the acquisition was primarily conducted on individually sampled plants with relatively simple backgrounds and limited canopy occlusion. Such conditions reduce the complexity of point cloud geometry compared to dense breeding fields, intercropping zones, or UAV-based large-scale acquisitions, potentially leading to optimistic model performance.

Second, the cross-sectional diameter measurement is based on ellipse fitting, which assumes that the stem exhibits relatively regular and near-cylindrical geometry. However, under natural field conditions, biological variations such as disease infection, irregular node swelling, stem curvature, and lodging can violate this assumption, resulting in unstable fitting or biased diameter estimation. Furthermore, although scaling and unit conversion were carefully calibrated, slight alignment deviations may still occur when comparing the automatically estimated diameters with manual measurements.

Third, although the proposed model was trained and validated on point clouds captured under actual field environments, its evaluation was limited to specific maize varieties and growth stages. In addition, the current dataset does not include dense canopy environments, overlapping foliage, or complex background interference such as weeds and soil residues, which are common in large-scale farming and high-throughput breeding trials. The generalizability of the method to other crop species, varying developmental phases, and large-scale phenotyping scenarios remains to be further explored. Additionally, natural background complexity and severe class imbalance in real farmland settings may introduce further challenges to segmentation robustness.Moreover, practical deployment in large-scale phenotyping settings may also require efficient sampling strategies, quality assessment mechanisms, and automatic rejection of low-fidelity scans.

Future work will focus on three major directions: (1) improving 3D reconstruction completeness and reliability under occlusion, wind-induced motion, illumination variability, and complex field backgrounds by integrating LiDAR, time-of-flight depth sensing, or depth-enhanced MVS; (2) enhancing model generalization across different species, developmental stages, and growth conditions through domain adaptation, multimodal fusion (RGB–depth–thermal), and semi-supervised learning; and (3) developing a portable field-deployable phenotyping system with automatic calibration, quality assessment, and real-time stem diameter estimation.Future investigations will also consider data acquisition strategies in densely planted and wind-disturbed farmland, enabling robust processing of partially incomplete or noisy point clouds. Additionally, we plan to incorporate biological shape priors and biomechanical deformation modeling to improve robustness under irregular growth, node swelling, and lodging conditions. These improvements will enhance scalability, robustness, and deployment potential for large-scale, in-field agricultural phenotyping.

## 5 Conclusion

This study introduces a three-dimensional measurement method for the stem diameter of maize during the jointing stage in a field environment. It proposes an improved PointNet++ network and combines a slice-ellipse structure feature fitting strategy to achieve high-precision measurement of the major axis and minor axis diameters of the stem in the main axis direction. The experimental results show that the proposed RLRSA-PointNet++ model achieves an intersection-over-union (IoU) of 0.980 and an accuracy rate of 0.943, enabling accurate segmentation of maize stems. This method achieved good prediction performance on 72 sets of maize samples, with average absolute errors of 1.27 mm and 1.38 mm for the major axis and minor axis, respectively, and determination coefficients of 0.87 and 0.82, respectively, verifying its adaptability and robustness in complex field environments.

This study provides a high-precision, generalizable technical path for the intelligent extraction of crop structural parameters in complex contexts, and has important application potential. This method not only provides technical support for the detailed monitoring and structural trait evaluation of maize phenotypes, but also provides key data support and methodological references for the exploration of the relationship between crop phenotypes and genotypes, as well as for intelligent breeding decisions.

## Data Availability

The raw data supporting the conclusions of this article will be made available by the authors, without undue reservation.
