# Supplementary material for: A 3D stem diameter measurement method for field maize at jointing stage: combining RLRSA-PointNet++ and structural feature fitting
Source: Front Plant Sci. 2026 Jan 12;16:1724096. doi: 10.3389/fpls.2025.1724096 (PMC12833335; doi:10.3389/fpls.2025.1724096)
Supplement: Supplementary file 1 [file Supplementaryfile1.docx]

Supplementary Material

This supplementary material provides additional technical details supporting the proposed method. It is organized into two main parts. First, we provide the pseudocode of the overall model architecture, demonstrating how the PointNet++ backbone is enhanced with Relative Position Encoding (RPE), Local Group Rearrangement Module(LGRM), Local Region Self-Attention (LRSA), and feature propagation mechanisms. This clarifies the feature flow, module integration strategy, and computational stages within the improved network.

Second, we provide the mathematical derivation of the Local Region Self-Attention (LRSA) mechanism. These details help further elucidate how LRSA leverages local geometric priors while maintaining the flexibility of self-attention to enhance structural feature learning.

# Forward Propagation Pipeline of the Proposed RLRSA-PointNet++ Network

**# 1: Set Abstraction Layers SA1–SA2**

Input: Raw point cloud P ∈ ℝ^{B×N×9} (XYZ, RGB, Normals)

1: l0_xyz ← P[:, :, 0:3]

2: l0_points ← P

3: (l1_xyz, l1_points) ← SA1(l0_xyz, l0_points)

*# Sampling: 1024 points, radius=0.1, group=32*

*# Output: l1_xyz ∈ ℝ^{B×1024×3}, l1_points ∈ ℝ^{B×64×1024}*

4: (l2_xyz, l2_points) ← SA2(l1_xyz, l1_points)

*# Sampling: 256 points, radius=0.2, group=32*

*# Output: l2_xyz ∈ ℝ^{B×256×3}, l2_points ∈ ℝ^{B×128×256}*

**#2: Relative Position Encoding (RPE)**

Input: l2_xyz ∈ ℝ^{B×256×3}, l2_points ∈ ℝ^{B×128×256}

5: center_xyz ← Mean(l2_xyz, dim=2, keepdim=True)  *# (B, 1, 3)*

6: rpe_feat ← RPE(l2_xyz, center_xyz)  *# Output: (B,128,256)*

7: l2_points ← l2_points + rpe_feat  *# Feature fusion*

**#3: Local Group Rearrangement (LGRM)**

Input: l2_points ∈ ℝ^{B×128×256}

8: grid_feat ← LGRM(l2_points)

*# Reshape unordered points into grid*

*# Output grid_feat ∈ ℝ^{B×128×16×16}*

**#4: Local Region Self-Attention (LRSA)**

Input: grid_feat ∈ ℝ^{B×128×16×16}

9: attn_feat ← LRSA(grid_feat)

*# Attention over local spatial blocks*

*# Output: ℝ^{B×128×16×16}*

10: l2_points ← Flatten(attn_feat)

*# Flatten spatial grid → sequential point features*

*# Output: (B,128,256)*

**#5: SA3 and SA4 - Higher Semantic Abstraction**

Input: l2_xyz ∈ ℝ^{B×256×3}, l2_points ∈ ℝ^{B×128×256}

11: (l3_xyz, l3_points) ← SA3(l2_xyz, l2_points)

*# Output: l3_xyz ∈ ℝ^{B×64×3}, l3_points ∈ ℝ^{B×256×64}*

12: (l4_xyz, l4_points) ← SA4(l3_xyz, l3_points)

*# Output: l4_xyz ∈ ℝ^{B×16×3}, l4_points ∈ ℝ^{B×512×16}*

**#6: Feature Propagation (FP4–FP1)**

13: l3p ← FP4(l3_xyz, l4_xyz, l3_points, l4_points)

14: l2p ← FP3(l2_xyz, l3_xyz, l2_points, l3p)

15: l1p ← FP2(l1_xyz, l2_xyz, l1_points, l2p)

16: l0p ← FP1(l0_xyz, l1_xyz, None, l1p)

**#7: Classification Head**

17: x ← MLP(l0p)  *# Conv1d + BN + ReLU + Dropout*

18: S ← Softmax(x) *# Output: (B×N×num_classes)*

19: return S

# Local Region Self-Attention (LRSA) Mathematical Formula Derivation

First, perform patch division and reconstruction. Let the input feature map be $\mathbf{X}\in{\mathbb{\mathbb{R}}}^{B\times C\times H\times W}$, use a sliding window method to divide it into several local patches. Each patch has a size of $P\times P$, with a sliding step size of $s$. After flattening all patches, a patch sequence is formed as shown in Equation (1), where $\mathcal{p}$ denotes the set of all patches; $\mathbf{P}_{i}$denotes the patch obtained in the i-th extraction; and N denotes the number of patches.

| $\mathcal{p=}{\{\mathbf{P}_{i}\in{\mathbb{\mathbb{R}}}^{C\times P\times P}\}}_{i=1}^{N}$ | (1) |
| --- | --- |

Where $N=\left\lceil\frac{H-P}{s} \right\rceil\times\left\lceil\frac{W-P}{s} \right\rceil$.

Each patch is stretched into a vector sequence for attention calculation, as shown in equation (2):

| $\mathbf{Z}_{i}=reshape(\mathbf{P}_{i})\in{\mathbb{\mathbb{R}}}^{P^{2}\times C}$ | (2) |
| --- | --- |

Then, multi-head self-attention mechanisms are used for local modeling within each patch. Let $\mathbf{Z}_{i}$ be the input. First, query (Q), key (K), and value (V) vectors are constructed, as shown in Equation (3):

| $\mathbf{Q}=\mathbf{Z}_{i}\mathbf{W}^{Q}$,$\mathbf{K}=\mathbf{Z}_{i}\mathbf{W}^{K}$,$\mathbf{V}=\mathbf{Z}_{i}\mathbf{W}^{V}$ | (3) |
| --- | --- |

Among them, $\mathbf{W}^{Q}$, $\mathbf{W}^{K}\in{\mathbb{\mathbb{R}}}^{C\times d_{k}}$, $\mathbf{W}^{V}\in{\mathbb{\mathbb{R}}}^{C\times C}$ are learnable parameters.

Update using scaled dot-product attention, as shown in equation (4):

| $Attention\left( \mathbf{Q,K,V} \right)=softmax(\frac{\mathbf{Q}\mathbf{K}^{\mathbf{T}}}{\sqrt{d_{k}}})\mathbf{V}$ | (4) |
| --- | --- |

The characteristics of each patch after updating are shown in Equation (5):

| $Z_{i}'=Attention(Q,K,V)+Z_{i}$ | (5) |
| --- | --- |

After attention processing, the features are restored to the original patch format and finally combined back into the original-size feature map through a reconstruction operation. To further enhance the local contextual semantic expression, this paper adds a deep convolutional feedforward network (ConvFFN) after attention. Given a feature vector $\mathbf{x}\in{\mathbb{\mathbb{R}}}^{h\times w\times C}$, the ConvFFN structure is as follows:

1. The fully connected transformation is shown in Equation (6):

| $\mathbf{z}_{1}=GeLU(\mathbf{x}\mathbf{W}_{1})$ | (6) |
| --- | --- |

1. Deep convolution enhancement, corresponding to formula (7):

| $\mathbf{z}_{2}=DepthwiseConv2D(\mathbf{z}_{1})+\mathbf{z}_{1}$ | (7) |
| --- | --- |

3) The output mapping is $\mathbf{z}_{out}=\mathbf{z}_{2}\mathbf{W}_{2}$, where $\mathbf{W}_{1}$ and $\mathbf{W}_{2}$ are fully connected weights.

Finally, the output features are shown in Equation (8):

| $\mathbf{X}^{out}=\mathbf{z}_{out}+\mathbf{x}$ | (8) |
| --- | --- |

Insert the LRSA module between the SA second and SA third grouping layers to fully utilize the spatial structural information contained in the middle layer semantic features. This module can effectively suppress background interference points and improve the accuracy and consistency of stem boundary recognition.
